# Systematic trends in water properties across Jacob's ladder density functionals

**DOI:** 10.1039/d6dd00571c

**Published:** 2026-09-25

**Authors:** Nam H. Đào, Daniel S. King, Frederick Stein, Xiaoyu Wang, Dongjin Kim, Gerit Brandenburg, Jürg Hutter, Bingqing Cheng

**Affiliations:** a Department of Chemistry, UC Berkeley California 94720 USA bingqingcheng@berkeley.edu; b Bakar Institute of Digital Materials for the Planet, UC Berkeley California 94720 USA king1305@berkeley.edu; c Department of Chemistry, University of Zurich Winterthurerstrasse 190 8057 Zurich Switzerland f.stein@hzdr.de; d Center for Advanced Systems Understanding (CASUS), Helmholtz-Zentrum Dresden-Rossendorf e.V. (HZDR) Bautzner Landstraße 400 01328 Dresden Germany; e Group Science and Technology Office, Merck KGaA Frankfurter Str. 250 Darmstadt 64293 Germany; f Chemical Sciences Division, Lawrence Berkeley National Laboratory Berkeley CA USA

## Abstract

Kohn–Sham density functional theory is the workhorse of computational materials science and chemistry, but the optimal choice of exchange–correlation approximation for a given system is often unclear. Traditional benchmark studies typically compare selected density functional approximations (DFAs) against high-level quantum chemistry results for small structures and static energies, rather than against experimental thermodynamic or response properties. Here, we show how machine learning interatomic potentials (MLIPs) allow for molecular dynamics simulations of these properties at scale, thereby providing a thermodynamic assessment for DFAs. Focusing on the well-studied but challenging case of bulk water at ambient conditions, we evaluate 11 DFAs spanning the top four rungs of Jacob's ladder (GGA, *meta*-GGA, hybrid, and double hybrid) with and without empirical dispersion corrections, using consistent basis set selections. Using MLIPs trained on each DFA, we compute infrared spectra, heat capacities, radial distribution functions, and diffusivities from classical molecular dynamics simulations. We find a general improvement up Jacob's ladder towards double hybrids, though there is substantial variation between DFAs within the same rung and across different properties. Dispersion corrections yield only a modest influence on the computed properties. We elucidate the similarities and hierarchy among DFAs, both based on correlating the DFA energies and forces for the same training configurations, and based on analyzing the finite-temperature observables. Our work thus demonstrates the power of MLIPs for finite-temperature benchmarking of electronic structure methods.

## Abstract 2

Kohn–Sham density functional theory is the workhorse of computational materials science and chemistry, but the optimal choice of exchange–correlation approximation for a given system is often unclear.

## Graphical abstract

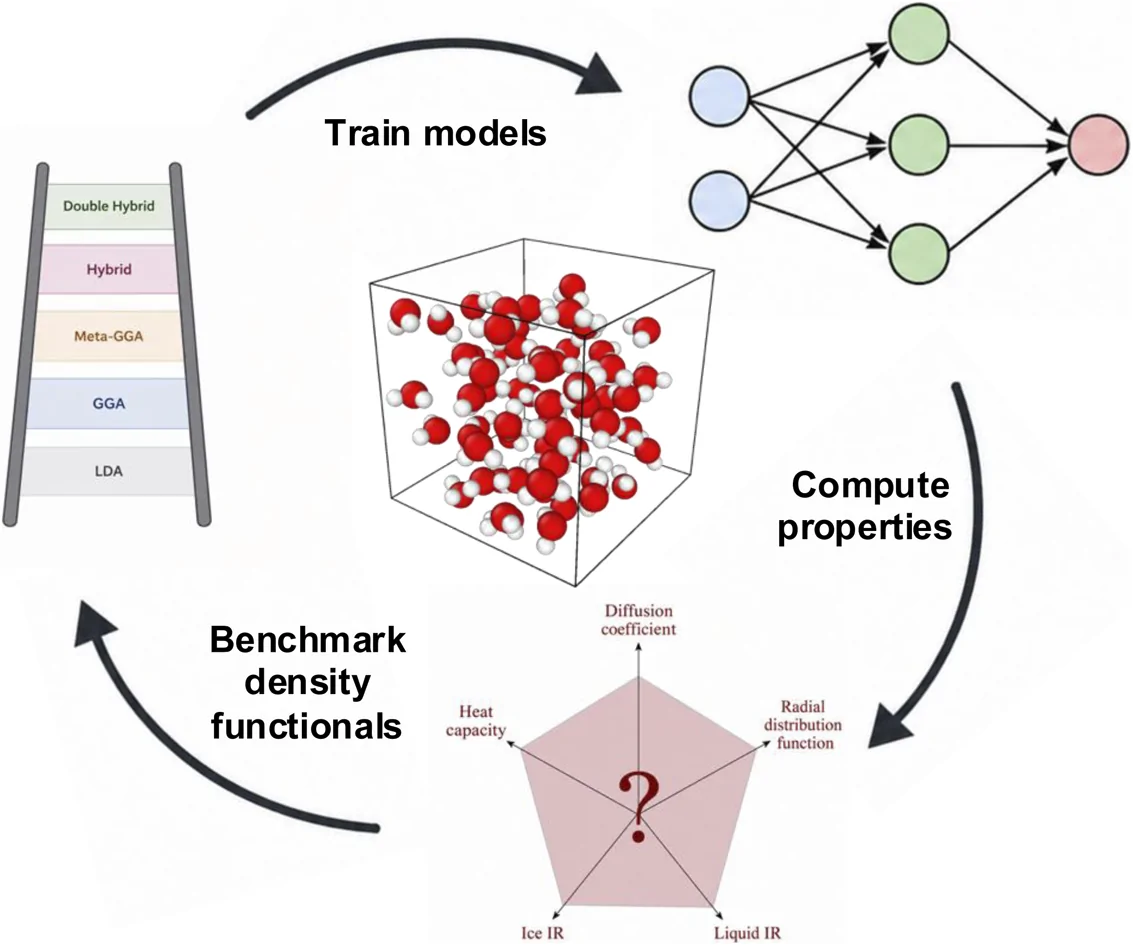

## 1 Introduction

When modeling a specific system of materials or molecules with Kohn–Sham density functional theory, it is necessary to select a suitable density functional approximation (DFA) to calculate the exchange–correlation energy. The accuracy of DFAs is typically classified *via* Perdew's “Jacob's ladder”^1^ in which higher rungs treat the calculation of exchange–correlation with increasing complexity, often improving accuracy but at greater computational cost.^1,2^ In practice, selecting a suitable DFA for a particular system is often based on intuition, conventions of the field, or better still, benchmark tests for similar systems. One type of benchmark is based on comparing computed DFA properties of selected small systems (typically less than a hundred atoms) to more accurate reference methods (*e.g.*, coupled cluster theory).^3^ However, going beyond such static properties of small systems in these benchmark studies is prohibitive due to the tremendous computational cost of the reference methods. For bulk amorphous systems such as water, benchmarking functionals is particularly challenging because of the large system sizes and the lack of translational symmetry.

The literature on benchmarking DFAs for water is extremely rich.^4–6^ A common approach to benchmarking DFAs for water involves using *ab initio* molecular dynamics (AIMD) to compute experimental observables (*e.g.*, radial distribution functions (RDFs), densities, *etc.*) at finite temperatures.^6–23^ Early work by Parrinello, Galli, and others showed that GGA functionals provide overstructured descriptions.^7,8^ This was followed by various studies showing that hybrid functionals such as B3LYP and PBE0 improve water RDFs and diffusivities relative to GGAs.^9,14,19^ Lee and Tuckerman^10^ were among the first to report AIMD infrared spectra, and also computed RDFs, diffusivity, and hydrogen bond lifetimes using BLYP. Pestana *et al.*^20^ showed that *meta*-GGAs can reproduce bulk water RDFs, diffusivity, and infrared (IR) spectra with comparable accuracy to hybrid functionals. The SCAN functional in the *meta*-GGA rung was found to yield good structural and dynamical properties for bulk water.^17,21,23^ A number of studies explored the role of dispersion and nonlocal van der Waals corrections, generally finding minor or no improvement.^11–13^ Recently, Villard *et al.*^6^ compiled a comprehensive comparison of DFAs on different rungs along with post-Hartree–Fock methods in computing water properties such as RDFs, density, and diffusivity, highlighting the increased accuracy found going up Jacob's ladder.

However, to our knowledge, no comprehensive assessment of double-hybrid functionals for liquid water exists outside a few isolated studies on gas-phase or small molecular clusters.^24,25^ Moreover, studies using AIMD can generally only afford to compute properties for a handful of functionals, leaving room for more extensive benchmarks. In addition, nuclear quantum effects (NQEs) are typically omitted in many AIMD benchmarks due to the computational cost of path-integral molecular dynamics (PIMD) simulations,^26^ although they influence the structure and dynamics of hydrogen-bonded liquids such as water.^26–29^

In the past two decades, machine learning interatomic potentials (MLIPs) have revolutionized atomistic modeling by learning atomic interactions from DFT to drive efficient molecular dynamics (MD) simulations. In this time, the training accuracy of MLIPs has also greatly improved thanks to advancements in the architecture: from the early Behler–Parrinello network based on atom-centered symmetry functions,^30^ to atomic cluster expansion methods,^31^ to message-passing graph neural networks,^32,33^ to MLIPs augmented by long-range electrostatics.^34,35^ Notably, long-range electrostatic MLIPs utilizing the Latent Ewald Summation (LES) approach^36–39^ can infer long-range electrostatics by training solely on energies and forces. LES thus enables facile computation of IR spectra by inferring Born effective charge (BEC) tensors without explicit training on such electrical response properties.^37^

Given this progress, it has become very popular to use MLIPs as surrogate models for computing the thermodynamic properties of systems based on certain DFAs. For water, there are numerous such studies. For example, Cheng *et al.*^40^ used MLIPs trained on a hybrid functional to predict melting points, density isobars, RDFs, and other thermodynamic properties of liquid and solid water. Monserrat *et al.*^41^ showed that a MLIP trained on liquid water can accurately predict ice densities, lattice energies, and vibrational properties across many ice phases. Schran *et al.*^42^ developed an automated workflow for constructing and utilizing MLIPs for complex aqueous systems. Zhang *et al.*^43^ systematically compared the performance of the SCAN functional to the hybrid SCAN0 for liquid water by training a Deep-Wannier neural network for running DPMD, providing trajectories for RDFs, diffusivity, and various electronic properties. Nevertheless, these earlier works have typically been restricted to a handful of DFAs. Moreover, many previous works used early-generation MLIPs, which can yield larger training errors which may not be negligible for the purpose of benchmarking DFAs.

Here, we benchmark DFAs using long-range electrostatic MLIPs trained on the energies and forces of various DFAs and use the trained models to compute thermodynamic and response properties. We focus on the well-studied yet challenging system of room-temperature water using 11 base density functionals using the cc-TZ basis set:^44–46^ 2 GGA functionals (optB88-vdW,^47^ PBE);^48^ 3 *meta*-GGA functionals (SCAN,^49^ TPSS,^50^ r^2^SCAN^51^); 4 hybrid functionals (B3LYP,^52,53^ HSE06,^54^ PBE0,^55^ revPBE0);^47,55^ and 2 double hybrid functionals (revDSD-BLYP,^24,56^ revDSD-PBEP86).^24,57^ Additionally, to investigate the effects of dispersion corrections and basis sets, we compute 17 augmented D3 (ref. 58) and D4 (ref. 59) versions of most functionals (PBE-D3, PBE-D4, SCAN-D3, SCAN-D4, TPSS-D3, TPSS-D4, r^2^SCAN-D4, B3LYP-D3, B3LYP-D4, HSE06-D3, HSE06-D4, PBE0-D3, PBE0-D4, revPBE0-D3, revPBE0-D4, revDSD-BLYP-D4, revDSD-PBEP86-D4) and additionally compare to revPBE0-D3 data computed with the TZV2P basis.^40,60–62^ As such, there are a total of 29 DFAs considered in this study. For each of these DFAs, we generate MD simulations with long-range electrostatic MLIPs to compute IR spectra, heat capacities, RDFs, and diffusivity.

Besides assessing the accuracy and behavior of these DFAs by benchmarking their predictions against experimental and previous simulated values of room-temperature bulk water, we explore the following questions: How are different functionals correlated? What are the influences from dispersion corrections? Is there a systematic benefit to going up Jacob's ladder?

## 2 Results

### 2.1 Comparison of energies and forces

The dataset consists of bulk liquid water configurations taken from Cheng *et al.*^40^ For each DFA without dispersion correction, we recompute the energies and forces for these configurations using CP2K with various DFAs. In general, the DFA predictions are dependent on the choice of the basis set, pseudopotentials, energy cutoffs, and other numerical settings. Here, we adopt the cc-TZ basis set^44–46^ with rung-optimized GTH pseudopotentials for all DFAs. The plane wave energy cutoff was set to 800 Ry. The D3 or D4 dispersion corrections were added afterward using the DFTD3 and DFTD4 packages.^59,63,64^ The original dataset^40^ was computed using the revPBE0-D3 functional with an energy cutoff of 400 Ry and using the TZV2P basis set.^60–62^ cc-TZ (3s3p2d1f for O, 3s2p1d for H) is more angularly complete than TZV2P (3s3p2d for O and 3s2p for H), and is used to put the double hybrids on equal footing to the lower three rungs, as wave function methods converge significantly slower than DFT with respect to basis set size.^46^ However, to investigate the influence coming from the choice of the basis set we maintain this original data in the benchmark set under the notation revPBE0-D3-TZV2P. More details on the construction of the dataset are provided in the Methods section.

First, we analyze the similarities and correlations between the 29 DFAs from the energy and force data of the training configurations. We quantify the similarity between DFAs by constructing a 29 × 29 correlation matrix whose entries1
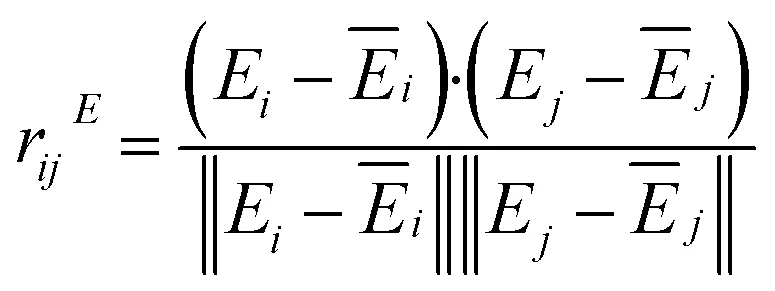
measure the Pearson correlation between the per-snapshot energy vectors of two DFAs (*i* and *j*), with an analogous matrix *r*^*E*^ built from stacked Cartesian force components. Principal component analysis (PCA) is then applied to each correlation matrix, so each point in the PCA plots corresponds to a DFA embedded according to its similarity to all other functionals. Fig. 1a and b show PCA projections of each DFA in the correlation matrix, demonstrating clear separation by rung. In both PCA plots, DFAs of the same rung stay close to each other in the latent space, with a systematic shift moving from GGAs to *meta*-GGAs to hybrids to double hybrids. The dispersion-corrected functionals (appended D3 (ref. 58) or D4 (ref. 59)) unsurprisingly output similar energies and forces as the parent functional. As such, the locations of these functionals on the PCA maps are generally very close to the original ones.

**Fig. 1:**
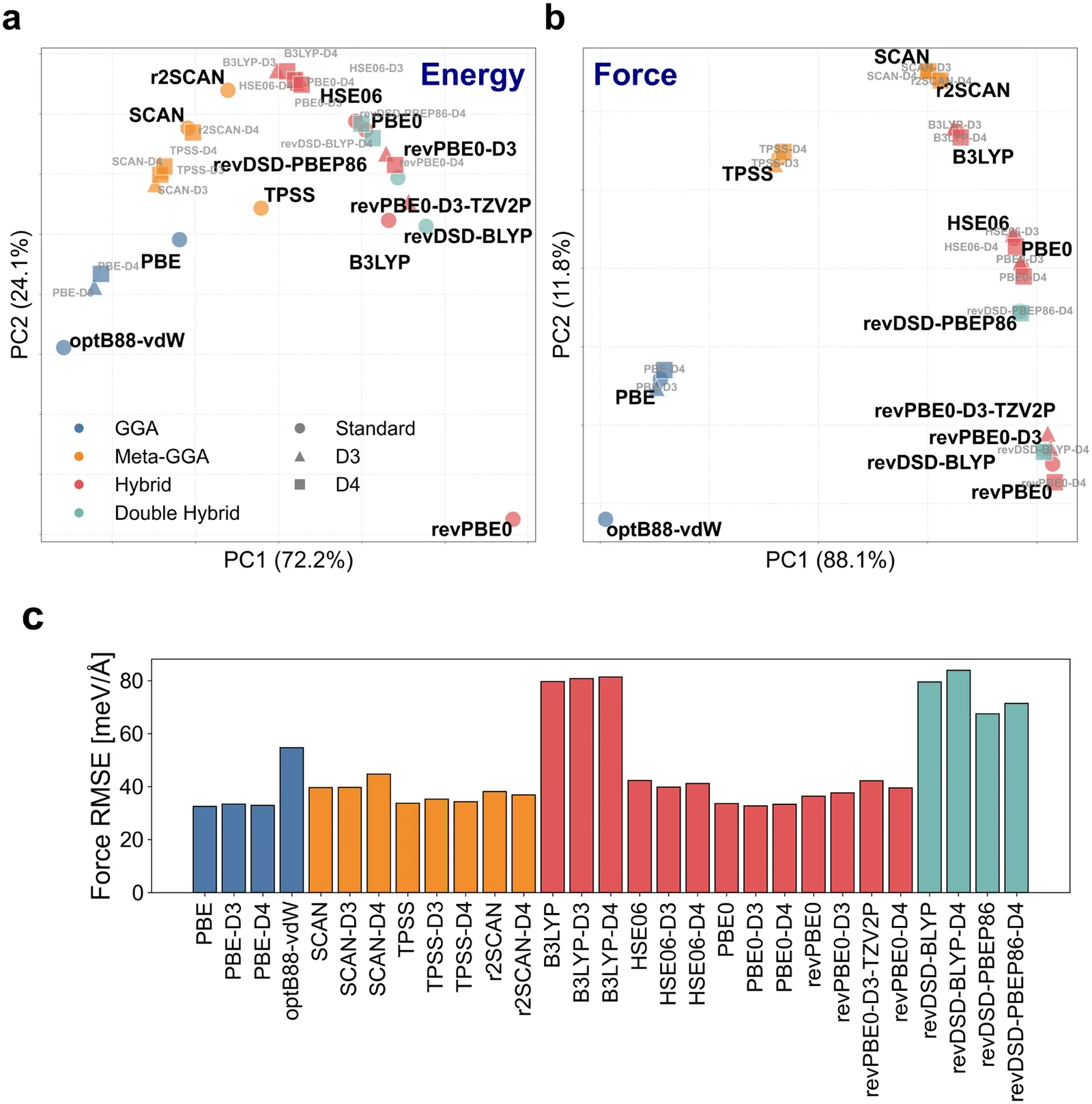
Comparison of predicted energies and forces of 1526 training configurations produced by density functionals across different rungs of Jacob's ladder. (a) The correlation of the DFA energies from principal component analysis (PCA). (b) The correlation of the DFA forces from PCA. (c) The root mean squared error (RMSE) of force predictions by CACE-LR for each DFA in meV Å^−1^.

These observed trends suggest that families and rungs can be distinguished by their energies and forces alone, hinting at systematic differences between DFAs that may propagate to macroscopic predictions. In what follows, we will analyze whether such systematic differences are also found in observables computed from the molecular dynamics of bulk water driven by MLIPs trained on the different DFAs.

The Cartesian Atomic Cluster Expansion (CACE)^65^ employs polynomial expansions of atom-centered clusters^31^ with high body orders. We use a MLIP architecture that combines CACE with LES augmentation,^36^ which we refer to as CACE-LR hereafter. The LES component in these CACE-LR models can predict BEC tensors,^37,38^ enabling straightforward IR spectra from MD trajectories without additional training labels.

Fig. 1c shows the training force root mean squared errors (RMSEs) of the CACE-LR models, demonstrating high fidelity to reference electronic structure calculations. Energy RMSE plots shown in Fig. S2 demonstrate similarly low errors on the order of 0.5–1.1 meV for all DFAs. We find that double hybrid density functionals and B3LYP are more difficult to fit (RMSE ≈ 79 meV Å^−1^), possibly reflecting increased noise in the underlying data. To quantify the impact of long-range effects on force errors, a comparison of RMSE errors using CACE and CACE-LR on all functionals is provided in the SI. Overall, CACE-LR typically slightly reduces force RMSE (2–5 meV Å^−1^) relative to the short-ranged CACE MLIPs.

We then use the fitted MLIPs to obtain NVT trajectories of a cubic cell containing 64 water molecules at 300 K at density *ρ* = 997 kg m^−3^. Details of the MD settings are provided in the Methods section.

### 2.2 Infrared spectra

The IR spectrum of liquid water is highly sensitive to both intermolecular hydrogen-bond dynamics and intramolecular vibrations.^72–74^Fig. 2a shows the IR spectra for all 29 DFAs and groups them by rung, illustrating the superior performance of double hybrids relative to experimental data. To approximate NQEs, band maxima from each DFA were red-shifted using literature-motivated values (5 cm^−1^ for *ν*_T_ and *ν*_L_, 60 cm^−1^ for *ν*_B_, and 175 cm^−1^ for stretching *ν*_S_) which are further elaborated on in the Methods section.^66–69^ To evaluate the validity of these shifts, we have performed thermostatted ring polymer molecular dynamics (TRPMD) simulations as reported in the SI, which show that our classical shifted peak positions agree well with explicity treatment of NQEs (Fig. S6).

**Fig. 2:**
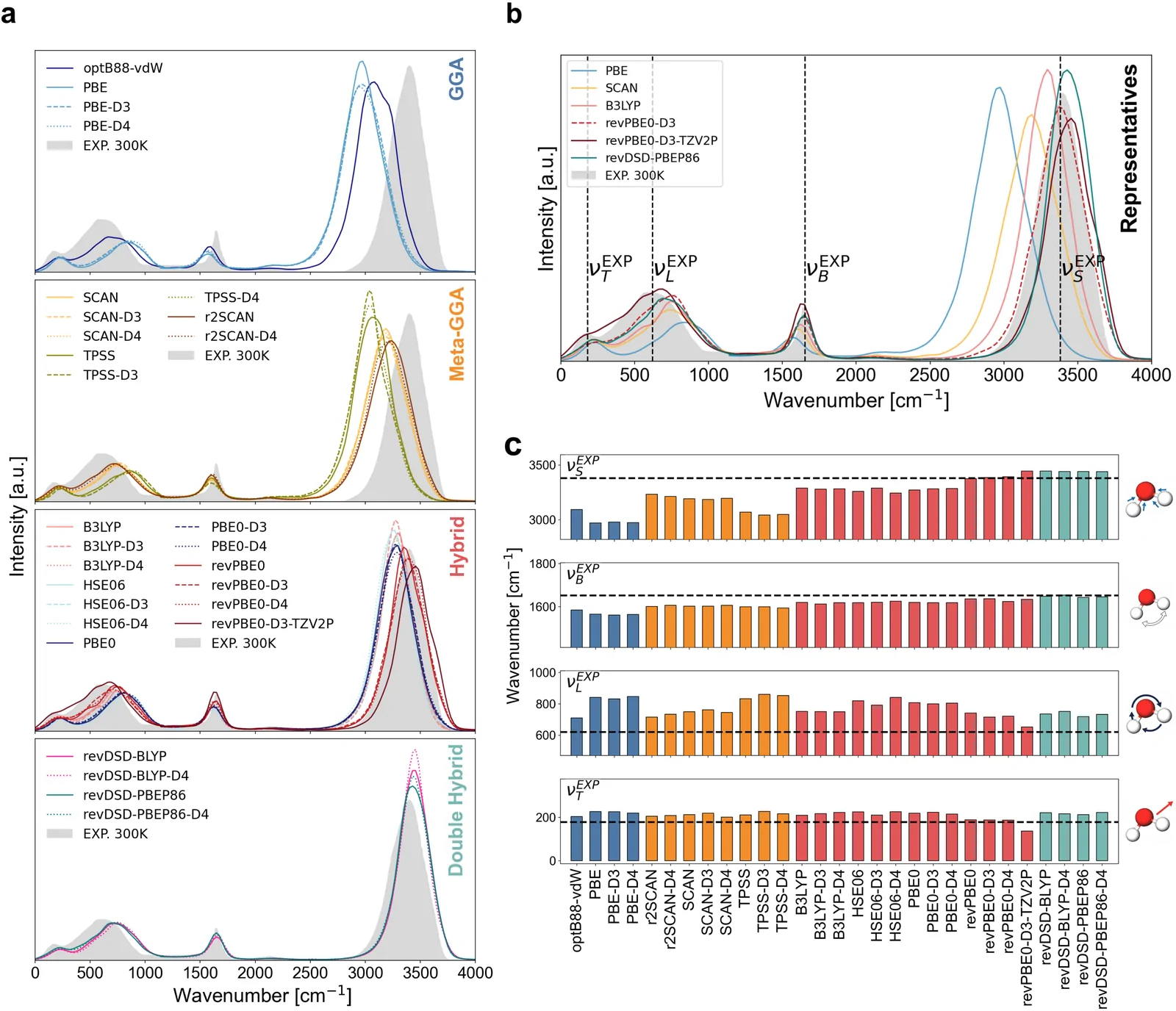
Infrared spectra (IR) of bulk water at 300 K and 1 g cm^−3^ computed by density functional approximations using Born effective charge tensors from long-range electrostatic machine learning interatomic potentials. The IR were then peak-shifted for each vibrational mode (translation *ν*_T_, libration *ν*_L_, bending *ν*_B_, and stretching *ν*_S_) to account for nuclear quantum effects using values from literature,^66–69^ which introduces discontinuities to the spectra. The experimental infrared spectra are provided for ref. 70 and 71. (a) IR of bulk water for all functionals separated by rung on Jacob's ladder. (b) IR of bulk water for select functionals with experimental peaks shown with dashed lines. (c) Comparison of all density functional vibrational modes *ν*_T_, *ν*_L_, *ν*_B_, and *ν*_S_ compared to the experimental values.^66–69^

To validate our approach, Table S3 provides a comparison of the computed peaks *ν*_T_, *ν*_L_, *ν*_B_, and *ν*_S_ with prior AIMD studies using revPBE0-D3.^20,67^ We find that our computed revPBE0-D3 peaks agree well with these results, with all computed modes within roughly 50 cm^−1^ of the previous studies. Use of the cc-TZ basis appears to result in mild blueshifting of the *ν*_T_ and *ν*_L_ bands and redshifting of *ν*_S_ relative to revPBE0-D3-TZV2P. The close agreement between our unshifted revPBE0-D3 peaks and the revPBE0-D3-TZV2P AIMD reference indicates that our MLIP-driven classical MD faithfully reproduces the underlying DFT-level vibrational structure prior to any NQE correction.

The translation bend *ν*_T_ shows little variation between DFAs individually and by rung, with values clustering around 201–227 cm^−1^ for almost all DFAs shown, which represents a general blue shift relative to experiment at *ν*_T_ = 178.2 cm^−1^.^70,71^ Likewise, *ν*_L_ shows little difference in performance between rungs, as functionals over-predict relative to experiment by roughly 32–220 cm^−1^, although double hybrids notably show the best performance on average.

In contrast, the bending and stretching modes *ν*_B_ and *ν*_S_ show a clear systematic improvement moving from rung to rung, with the NQE-shifted double-hybrid peaks in nearly exact agreement with experiment. Notably, we find that *ν*_B_ and *ν*_S_ frequencies are strongly correlated (Pearson coefficient *r* = 0.95, Fig. S13). This correlation makes sense as these higher frequency modes both depend mainly on intramolecular degrees of freedom. Across all density functionals and vibrational modes, D3 and D4 corrections generally provide little to modest improvement relative to experimental *ν*.

To further investigate trends between DFT rungs, we have also computed the IR spectrum of hexagonal ice, which is shown in Fig. S7. We find that these results largely complement those of liquid water. In particular, the intramolecular bending and stretching modes exhibit strong correlations across phases (*ν*_B_*vs.ν*_B_^Ih^, *ν*_S_*vs.ν*_S_^Ih^ with *r* = 0.76, *r* = 0.97 respectively), indicating that the description of these modes by DFAs largely translates from liquid to solid.

### 2.3 Heat capacity

Molar heat capacity was computed from total energy fluctuations using 
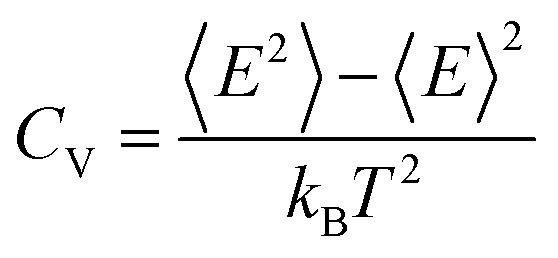
. To provide a consistent classical reference that avoids assuming functional-independent NQEs, we benchmark our computed *C*_V_ values against the classical MB-pol result of 116.5 J (mol^−1^ K^−1^)^75^ as the primary reference, with the experimental value^76,77^ reported as a secondary comparison. By comparing simulated results against experimental measurements (*C*_V_ ≈ 74.5 J (mol^−1^ K^−1^)),^77^ path-integral molecular dynamics (PIMD),^78^ and data-driven many-body potential (MB-Pol) references,^75^ we evaluate how accurately different DFAs capture water's thermodynamic behavior and the effects of dispersion contributions on these results.

The top plot in Fig. 3c shows the *C*_V_ computed for all density functionals, where the DFAs largely overestimate experimental *C*_V_. This systematic overestimation of heat capacity is in line with classical SPC/E simulations.^82^ This supports the consensus that *C*_V_ at 300 K is strongly quantum in character, so explicit PIMD is needed to reach the experimental baseline, particularly for thermodynamic properties.^83–85^ However, functional predictions clearly cluster around the classical MB-pol value,^75^ which we take as a classical water reference.

**Fig. 3:**
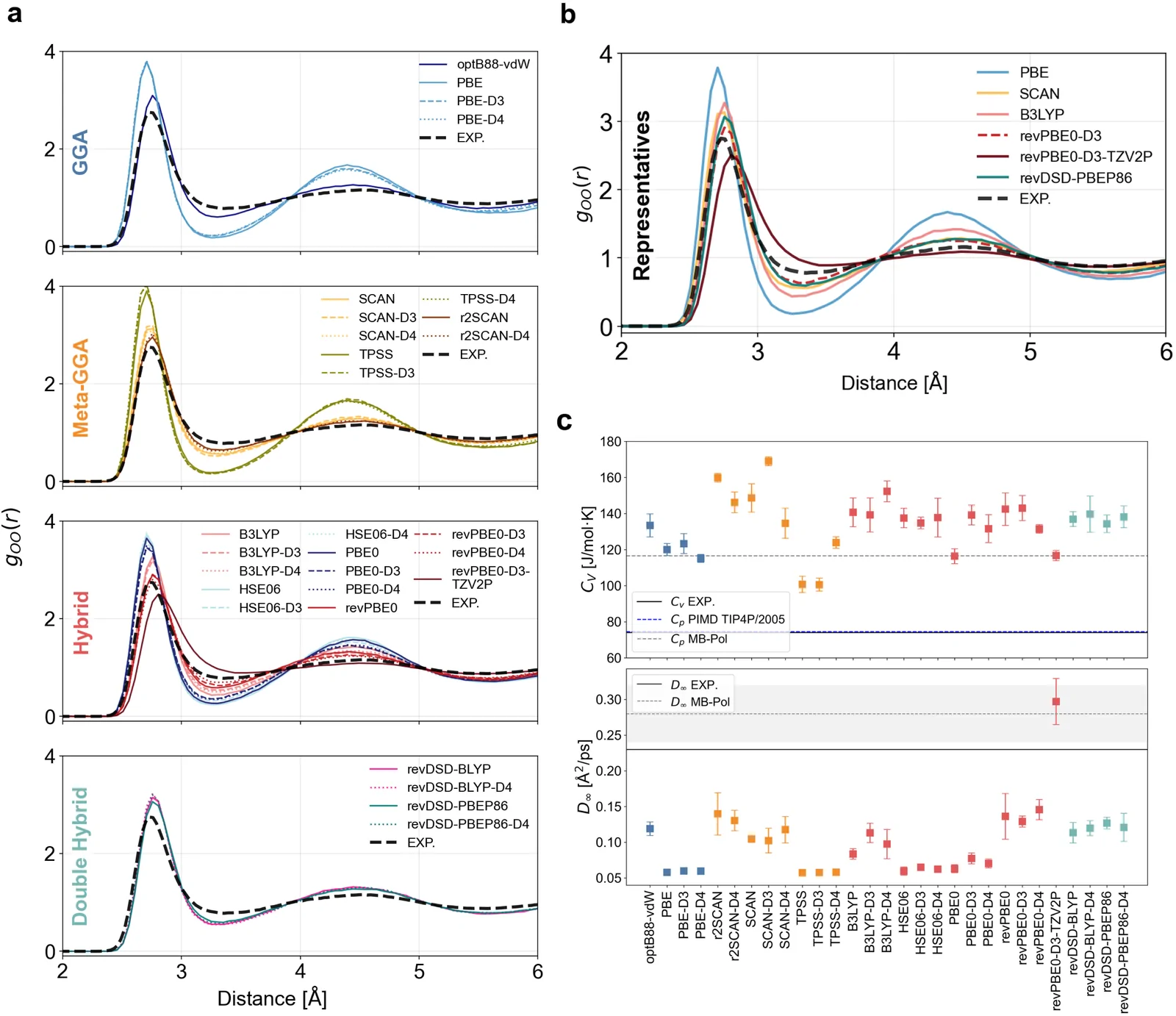
Structural, thermodynamic, and dynamic properties of water. (a) Oxygen–oxygen radial distribution functions *g*_OO_(*r*) for density functionals categorized by rungs of Jacob's ladder. The experimental *g*_OO_(*r*) from Soper^79^ is provided as reference. (b) *g*_OO_(*r*) for selected functionals. (c) Molar heat capacity *C*_V_ generated by density functionals, reported as classical values without nuclear quantum effect corrections. Experimental *C*_V_,^76,77^ path integral molecular dynamics using the TIP4P/2005 potential,^78^ and MB-Pol^75^ values (using the approximation *C*_P_ ≈ *C*_V_) are shown for reference. Computed self-diffusion coefficients of water *D*_∞_ utilizing the Yeh–Hummer finite-size correction.^80^ The MB-Pol classical value *D*_∞_ = 0.280 ± 0.04 Å^2^ ps^−1^ (ref. 75) and the experimental value *D* = 0.23 (ref. 81) are shown for reference. Error bars were estimated for both properties using block averaging with 5 blocks.

To validate our approach, we have compared our computed PBE heat capacity to AIMD from Pascal *et al.*^86^ We find that our uncorrected MLIP MD result is higher than the AIMD value (*C*_V_ = 120.0 *vs.* 89.3 J (mol^−1^ K^−1^)).^86^ However, we partly attribute this discrepancy to sampling time, as Pascal *et al.*^86^ utilized a 10 ps windows of simulation time totaling 150 ps using 128 water molecules. Computing *C*_V_ from the first 200 ps of simulation time, our MD results give a value of 83.0 J (mol^−1^ K^−1^), only slightly below the reference AIMD-PBE value. This difference also likely results from the use of the cc-TZ basis and the different DFT settings employed in this work.

Evaluating trends between functionals, we see broad agreement with the classical MB-pol value across all rungs, but with significant variance in predictions within the *meta*-GGA rung. This variance is largely due to differences between the SCAN family of functionals, which significantly overestimate the capacity, and the TPSS functionals, which generally underestimate the value. Again, the inclusion of dispersion generally yields only modest influence on the computed properties, beyond a few edge cases (*e.g.*, PBE0 *vs.* PBE0-D3/D4 and SCAN *vs.* SCAN-D3/D4).

### 2.4 Radial distribution function

The radial distribution function is a primary structural descriptor of liquid water, capturing the spatial correlations that define hydrogen-bond organization and local ordering. The RDF of the O–O bond distances is visualized for all density functional approximations in Fig. 3 compared to neutron diffraction results.^79^

Across all functionals, the first peak occurs near *r*_1_ ≈ 2.71–2.81 Å, in close agreement with diffraction-derived experimental peak positions values of 2.74 Å,^79^ while the peak height varies substantially by rung. The strong performance of DFAs in reproducing *g*_OO_(*r*) using classical nuclei is supported by literature, which demonstrates that NQEs have relatively little influence on bulk water *g*_OO_(*r*).^83,87^ Classical MB-pol results have also shown strong agreement with experiments at 298 K.^88^ NQEs do not play a significant role in the oxygen–oxygen RDFs, as shown in Fig. S5 that compares the classical and TRPMD *g*_OO_(*r*) across the five representative functionals.

To validate our approach, we additionally compare our predicted *g*_OO_(*r*) to various AIMD studies compiled by Villard *et al.*^6^ in Table S2. While our predicted *g*_OO_(*r*) generally reproduces the trends found in previous studies, we find mild but systematic overprediction of the predicted first shell peak height *g*_OO_(*r*_1_) (*i.e.*, overstructuring) across most functionals. This overstructuring appears to be due to the cc-TZ basis set and DFT settings used in this work, as our revPBE0-D3-TZV2P results (*g*_OO_(*r*_1_) = 2.51) closely match the reported AIMD value (*g*_OO_(*r*_1_) = 2.58). Thus, use of correlation-consistent basis sets seems to play a significant role in predicted *g*_OO_(*r*), although trends between functionals appear to largely be conserved.

Indeed, we find that the oxygen–oxygen radial distribution functions *g*_OO_(*r*) generally improve as the DFAs ascend up Jacob's ladder. GGA curves display higher first peaks and deeper first minima, characteristic of the overstructured water found in many PBE-based simulations.^89^ Likewise, the first and second-shell peak heights *g*_OO_(*r*_1_) and *g*_OO_(*r*_2_) progressively decrease across Jacob's ladder toward the experimental reference. SCAN-family and related *meta*-GGAs tighten *r*_1_ and moderate peak heights *g*_OO_(*r*_1_), providing better agreement with experiment. Looking at the second peak *g*_OO_(*r*_2_), *meta*-GGAs tend to populate interstitial waters more, softening the minimum between shells and slightly shifting *r*_2_.

Hybrids generally improve upon over-structuring further, but show mixed performance on the RDF second shell. In particular, the PBE0 and HSE06 families produced over-structured predictions of water that are closer to GGA and *meta*-GGA functionals (estimating *g*_OO_(*r*_1_) in the range 3.48–3.76 and *g*_OO_(*r*_2_) in the range 1.45–1.62). Thus, we find that double hybrids are the only group of functionals that demonstrate strictly better performance than GGAs.

Despite this, certain lower-rung functionals demonstrate remarkable performance in predicting *g*_OO_(*r*). In particular, r^2^SCAN places *g*_OO_(*r*_1_) close to the experimental value at 2.96 (experiment: 2.76) and reproduces a second-shell shape and amplitude that compare favorably to those of the double hybrids.

Again, we find that D3 and D4 corrections only yield modest influence. For example, the PBE family *g*_OO_(*r*_1_) increases by about 0.03–0.09 from parent to D3/D4 while keeping *r*_1_ and *r*_2_ unchanged, which represents a relatively minor shift. However, one notable exception is the PBE0 family, where the D3/D4 corrections reduce over-structuring of the parent functional by about 0.15–0.18 for *g*_OO_(*r*_1_) and about 0.11 for *g*_OO_(*r*_2_).

Averaging the errors in *g*_OO_(*r*_1_) and *g*_OO_(*r*_2_) for all DFAs by rung reveals the expected trend: higher-rung methods (especially double hybrids and the better-behaved hybrids such as r^2^SCAN-based and revPBE0-based variants) produce systematically smaller deviations from experiment, whereas lower-rung GGAs and certain *meta*-GGAs (*e.g.*, TPSS and TPSS-D3/D4) exhibit the largest over-structuring errors in peak heights (Fig. S4). Meanwhile, peak positions *r*_1_ and *r*_2_ remain relatively constant across all functionals. Fig. 3b shows the relative performances of representative functionals across rungs, demonstrating these trends.

### 2.5 Diffusivity

Diffusivity reflects the mobility and hydrogen bond dynamics of liquid water. Self-diffusion coefficients were computed using the Einstein relation relating the diffusivity *D* to the mean squared displacement (MSD). To account for finite-size effects, we apply the Yeh–Hummer correction^80^ to the diffusivities to yield *D*_∞_ of large systems.

Table S2 provides a quantitative comparison of MLIP MD generated *D*_∞_ compared to prior AIMD works. Functionals optB88-vdW,^6,23^ SCAN,^6,22^ PBE0,^6,14^ and revPBE0-D3-TZV2P^6,20^ all generally reproduce predictions of *D*_∞_ compared to their corresponding AIMD results reported in literature, with any differences likely to be partially due to differences in the basis sets employed. Full details are available in the Methods section.

The bottom plot of Fig. 3c shows the Yeh–Hummer corrected *D*_∞_ as computed by all DFAs and grouped by rung. As is seen, nearly all functionals considerably underestimate the experimental value near 0.23 Å^2^ s^−1^ (ref. 81) at 300 K, even after Yeh–Hummer corrections. This is in line with previous AIMD works.^6,13,17^ For example, Chen *et al.*^17^ showed that PBE drastically underestimates the self-diffusion coefficient relative to experimental results, even at a heightened temperature of 330 K in order to mimic the impact of NQEs. Here, we take as a reference the MB-Pol value of *D*_∞_ = 0.28 ± 0.04 Å^2^ ps^−1^ at 298 K (ref. 75) computed using classical MD, which somewhat overestimates the experimental self-diffusion coefficient.

Interestingly, we find that both the first peak height *g*_OO_(*r*_1_) and the second peak height *g*_OO_(*r*_2_), exhibit a strong negative correlations with *D*_∞_ (*r* = −0.84, and *r* = −0.82), consistent with the link between over-structured water and low diffusivity.^90^ This correlation is most evident for PBE, TPSS, HSE06, and PBE0, which produce over-structured predictions of *g*_OO_(*r*_1_) and *g*_OO_(*r*_2_) and simultaneously low diffusivities *D*_∞_.

## 3 Discussion and conclusion

In this work, we have shown how state-of-the-art MLIPs allow for the thermodynamic benchmarking of DFAs. Focusing on the well-studied but challenging system of bulk water at room temperature, we utilized long-range models to compute several structural and response properties: infrared spectra (modes *ν*_T_, *ν*_L_, *ν*_B_, *ν*_S_), the heat capacity *C*_V_; radial distribution functions (*r*_1_, *r*_2_, *g*_OO_(*r*_1_), *g*_OO_(*r*_2_)); and the self-diffusion *D*_∞_. Notably, the use of MLIPs allowed for the benchmarking of double hybrid functionals for many of these properties.

The primary finding of our work is that there is a clear and systematic separation of DFAs by their respective rungs on Jacob's ladder, both in the energies and forces of the configurations as well as in the eventual computed properties (Fig. 1a). Systematic improvement up Jacob's ladder is most evident in improved IR bending and stretching modes (Fig. 2c), decreased overstructuring of *g*_OO_(*r*) (Fig. 3b), and improved estimation of *C*_V_ (Fig. 3c). This stratification suggests that the theoretical improvements incorporated into higher-rung functionals indeed translate into tangible improvements for thermodynamic and response properties in water.

To emphasize this systematic trend, Fig. 4a first normalizes all numerical features computed by the DFAs, then projects these properties onto their principal components *via* PCA. As is seen, this projection largely captures the same inter-rung clustering shown in the energies and forces of Fig. 1. Although some significant intra-rung variation exists, most notably in the performance of different *meta*-GGAs (*e.g.*, SCAN *vs.* non-SCAN DFAs), it is clear that the thermodynamic, structural, and response properties computed in this study largely distinguish DFAs by rung. Additionally, we find that outside of a few edge cases (*e.g.*, the computed *C*_V_ of PBE0/SCAN or the improvement of *ν*_L_ by revPBE0-D3 relative to revPBE0), dispersion corrections yield only modest influence. This is seen in Fig. 4a, as D3 and D4 variants generally cluster close to their parent functionals.

**Fig. 4:**
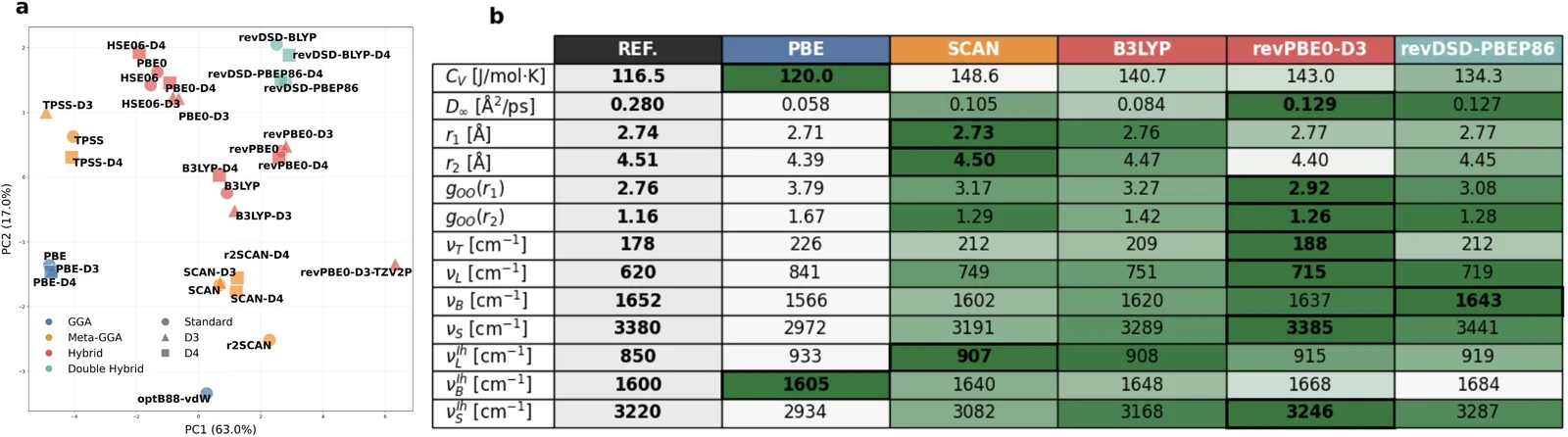
Comparison of all density functionals on all properties computed in this work. Properties include bulk water vibrational modes *ν*_T_, *ν*_L_, *ν*_B_, *ν*_S_; ice modes *ν*_L_^Ih^, *ν*_B_^Ih^, *ν*_B_^Ih^; heat capacity *C*_V_; RDF features *r*_1_, *r*_2_, *g*_OO_(*r*_1_), *g*_OO_(*r*_2_); and finite-size corrected self-diffusion *D*_∞_. (a) Principal Component Analysis (PCA) of all properties for all density functional approximations studied in this work. (b) Predicted values of each property for the representative functionals from each rung of Jacob's ladder, with experimental references shown in the leftmost column. Cells are colored by row: darker green indicates smaller absolute deviation from the reference value for that property across the five functionals PBE, SCAN, B3LYP, revPBE0-D3 and revDSD-PBEP86. For the heat capacity *C*_V_ and diffusion coefficient *D*_∞_, the reference are the classical MB-Pol values 116.5 J (mol^−1^ K^−1^) and *D*_∞_ = 0.280 ± 0.04 Å^2^ ps^−1^, respectively.^75^ The best-performing rung for each property is bolded.

Fig. 4b further highlights the performance of representative functionals from each rung on Jacob's ladder (PBE, SCAN, B3LYP, revPBE0-D3 and revDSD-PBEP86). For each property, the predicted value of each representative functional is reported alongside the experimental reference (or the classical MB-Pol values in the case of *C*_V_ and *D*_∞_). Cells are shaded by absolute deviation from the reference, with the color scale computed per-property: for each row, the functional closest to the reference is shown in the darkest green and the one furthest from the reference in white, with intermediate cells linearly interpolated between these two extremes. As expected, we see a gradual improvement in performance across rungs. Notably, we also observe that revPBE0-D3 performs comparably to and in some cases slightly better than the double hybrid revDSD-PBEP86 as shown in Fig. 4b.

Nevertheless, in spite of these trends, we emphasize that this work does not make a concrete recommendation concerning the use of a specific functional/rung for calculating the properties of water. Instead, the observed trends only serve to highlight broad differences between functionals when a consistent basis is used between rungs to compute a broad range of properties. In particular, our use of the more angularly complete cc-TZ basis sets departs from most water studies, and results evidently vary significantly depending on the basis set employed. Additionally, intra-rung variation can often be as significant as inter-rung variations for specific observables.

In summary, this work demonstrates the power of modern MLIPs for benchmarking DFAs on thermodynamic properties. These benchmarks provide a good sense of the trade-offs between different functionals and the effectiveness of MLIPs in interrogating these tradeoffs, with broad improvement up Jacob's ladder across several computed properties with modest effects from dispersion corrections. These findings are in line with previous AIMD works: GGAs such as PBE notoriously provide overstructured descriptions^7–9^ which are improved by the inclusion of exact exchange^14,19^ or particular *meta*-GGAs such as SCAN.^17,23^ Likewise, the effects of dispersion corrections have generally been found to be relatively minor and dependent on the underlying functional.^11,13^

We also want to highlight the limitations of our work. First, our results have dependence on the basis set and pseudopotentials: the same cc-TZ basis set and PBE0-optimized pseudopotentials were used throughout for consistency, but these were optimized for the performance of the higher-rung functionals. The significant effect of the higher-rung DFT settings is seen most clearly in this work between the performance of revPBE0-D3 and revPBE0-D3-TZV2P, in which the TZV2P results are found to give better values for most observables (Fig. 4b) but notably deviates in its prediction of the first peak radius *r*_1_ in the oxygen–oxygen radial distribution function.

Second, the NQEs are treated in an approximate manner in this work. For oxygen–oxygen radial distribution functions, we assumed that classical MD simulations are sufficient, which is well-supported by comparison with explicit TRPMD simulations reported in the SI (Fig. S5). For the IR spectra, we employed a peak-shifting scheme of the vibrational modes. This is also validated by the direct comparison between classical and TRPMD IR reported in the Fig. S6 of the SI. However, we note the obvious limitation of this approach including the discontinuity of the spectra. On the other hand, direct TRPMD has intrinsic limitations as an imaginary-time method. For other observables such as the heat capacity *C*_V_ and self-diffusion coefficient *D*_∞_, NQEs can be harder to quantify rigorously and may vary non-trivially across rungs of Jacob's ladder, so we instead based the comparison on classic values in this work.

Third, the training configurations for all DFAs remain fixed and were collected from revPBE0-D3 simulations at a narrow density range. This may introduce a bias against functionals that produce configurations that are dissimilar with the revPBE0-D3 ones, although we have performed generalizability test (Fig. S3 and S4) to show that the MLIPs are not extrapolating out of distribution during production MD. Nevertheless, more diverse configurations may be needed for probing a wider range of water properties such as density isobars, interfaces and phase diagrams.

## 4 Methods

### 4.1 Dataset

The training dataset contains DFT energy and force predictions on snapshots of a 64-molecule bulk water,^40^ totaling 1526 uncorrelated configurations.

The 11 separate density functionals used in this work span Jacob's ladder with 29 total DFAs (which include D3 and D4 dispersion corrected forms, as well as revPBE0-D3 using the original TZV2P basis). There are 4 GGA DFAs (optB88-vdW,^47^ PBE,^48^ PBE-D3, PBE-D4); 8 *meta*-GGA (SCAN, SCAN-D3, SCAN-D4, TPSS,^50^ TPSS-D3, TPSS-D4, r^2^SCAN,^51^ r^2^SCAN-D4); 13 hybrid (B3LYP,^52^ B3LYP-D3, B3LYP-D4, HSE06,^54^ HSE06-D3, HSE06-D4, PBE0,^55^ PBE0-D3, PBE0-D4, revPBE0,^91^ revPBE0-D3, revPBE0-D3-TZV2P, revPBE0-D4); and 4 double hybrid DFAs (revDSD-BLYP,^24^ revDSD-BLYP-D4, revDSD-PBEP86,^24^ and revDSD-PBEP86-D4).

For the revPBE0-D3 functional, two datasets of energy and force were available: revPBE0-D3-TZV2P, taken from prior work,^40^ and revPBE0-D3, which was computed for this study along with all other density functionals. This latter set of DFT data was computed using CP2K (ref. 92 and 93) using uniform convergence parameters, basis sets, and planewave cutoffs to ensure comparability. Comparing the two revPBE0-D3 datasets, the revPBE0-D3-TZV2P data was computed using the TZV2P basis set with PBE-optimized pseudopotentials. To represent the density, we use an auxiliary plane wave cutoff of 400 Ry, and the self-consistent field cycle is converged with a tolerance of *ε*_SCF_ = 5 × 10^−7^. Other computational details of the calculations are the same as those used in Marsalek *et al.*^67^

All DFT calculations were computed using a cc-TZ basis set with rung-separated pseudopotentials (PBE-optimized for GGA, SCAN-optimized for *meta*-GGA, and PBE0-optimized for hybrid/double hybrid functionals). This dataset also uses a denser real-space grid with a cutoff of 800 Ry and a stricter convergence parameter of *ε*_SCF_ = 1 × 10^−6^. Additional differences include the use of a Coulomb truncation correction in the newer calculations, whereas the original dataset relied on looser energy cutoffs and did not include these adjustments. Auxiliary density purification was also omitted in the updated dataset due to technical constraints, though its influence on thermodynamic observables is minimal.^94^ The CP2K input files used to generate the DFT data are available in the accompanying online repository.

To judge the generalizability of our dataset and models to dynamics simulations, we calculated energies and forces for 100 uncorrelated snapshots from the representative DFAs PBE and revPBE0. For both functionals, we find the force errors to be in line with their validation errors (SI Fig. S3 and S4), suggesting that our MLIPs are not extrapolating out of distribution during production MD.

### 4.2 MLIP training

CACE-LR models are trained on each DFT dataset, enabling a direct comparison of the short-range and long-range architectures. For the CACE representation, we used 6 Bessel radial functions with *c* = 12 and a cutoff of *r*_cut_ = 5.5 Å; the representation employed *N*_embedding_ = 3, *l*_max_ = 3, with *ν*_max_ = 3 and 1 layer of message passing (*T* = 1). For the long-range component, we use a 1-dimensional *q*, *σ* = 1.5 Å and d*l* = 3 Å. Final trained CACE-LR models averaged a validation RMSE of 0.74 meV per atom and 48 meV Å^−1^ for energies and forces, respectively.

### 4.3 MLIP MD

We propagate MLIP-driven classical NVT MD simulations at 300 K for bulk water and 100 K for ice Ih using the Atomic Simulation Environment (ASE) package.^95^ For each CACE-LR DFA, three sets of MD trajectories are propagated, all employing the Nosé–Hoover thermostat.^96,97^ The first two are MD trajectories of liquid bulk water at 300 K, one with a time step of 1 fs and propagated for 1 ns with an equilibration time of 50 ps, used to capture the macromolecular thermodynamic properties: heat capacity, diffusivity, and radial distribution functions. A second set of trajectories uses a smaller time step of 0.25 fs following 25 ps of equilibration and lasts 200 ps, where the smaller time step is used to capture the vibrational modes to be computed during IR spectra calculation. Finally, the third MD trajectory with 25 ps of equilibration and a run time of 200 ps is propagated for hexagonal ice Ih at 100 K using a 0.25 fs time step, used to compare the IR spectra of ice as computed by various DFAs. To assess finite-size effects, we additionally run 2 × 2 × 2 supercell (512-molecule) MD trajectories at 300 K with a 1 fs time step for 1 ns on the five representative functionals, with results provided in the SI. Finally, to explicitly assess NQEs, we run TRPMD simulations on the same five representative functionals for both liquid water observables and IR spectra, with results provided in the SI.

The 64-molecule starting configuration used in bulk water MD trajectories has side length *L* = 12.42 Å and density *ρ* = 997 kg m^−3^. The 64-molecule starting configuration of Ih ice was generated using GenIce2 (ref. 98) with density *ρ* = 0.8191 g cm^−3^.

### 4.4 Infrared spectra

The BEC is obtained as the derivative of the generalized polarization expression predicted from the LES charges.^37^ The IR spectrum is obtained by building the time-dependent polarization current *J*(*t*), and then computing the Fourier transform of the corresponding real-time current–current autocorrelation function:^37^2
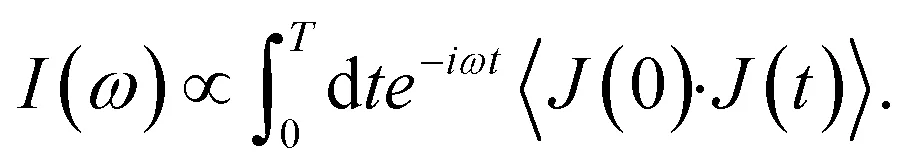


To account for NQEs, we apply band-dependent frequency shifts to the simulated IR spectra. The spectra are partitioned into vibrational regions corresponding to translational (0–300 cm^−1^), librational (300–1200 cm^−1^), bending (1200–2000 cm^−1^), and stretching (2800–3800 cm^−1^) modes of water, and each region is shifted independently.

Specifically, a nominal redshift of 5 cm^−1^ is applied to the low-frequency translational band *ν*_T_ and to the libration band *ν*_L_ consistent with reports that NQEs produce only minor frequency changes in these modes.^66,67^ For the intramolecular modes, a redshift of 60 cm^−1^ is applied to the bending band *ν*_B_ (ref. 68) and 175 cm^−1^ to the O–H stretching band *ν*_S_;^68,69^ these values fall within commonly used correction ranges for emulating NQEs in classical or semi-classical spectra. We retain these empirical band-dependent shifts as our primary NQE correction because they allow direct comparison to experiment while avoiding the spurious peak broadening and artificial redshifting of intramolecular modes associated with TRPMD.^99,100^ To validate this choice, we have run TRPMD simulations with our MLIP DFAs on representative functionals (see Fig. S6) and find that the peak locations of our empirically shifted classical spectra agree well with the explicit TRPMD results for the peaks *ν*_T_, *ν*_L_, *ν*_B_ and *ν*_S_, supporting the use of these shifts as first-order NQE corrections for these vibrational modes.

Similarly, we apply shifts to the hexagonal ice IR spectra. Specifically, a small redshift of 5 cm^−1^ is applied to the libration band *ν*_L_^Ih^, a moderate redshift of 18 cm^−1^ to the bending band *ν*_B_^Ih^ (taken from Rossi *et al.*^101^), and a larger redshift of 215 cm^−1^ to the OH-stretching band *ν*_S_^Ih^, consistent with explicit quantum-classical differences reported for ice Ih in the stretching region.^102^ A hexagonal ice IR spectra plot analogous to Fig. 2 is provided in the SI. The translational band is omitted due to a lack of a single, well-defined experimental maximum suitable for one-to-one peak alignment and comparison.^103^

### 4.5 Heat capacity and diffusivity

For the heat capacity *C*_V_ and diffusion coefficient *D*_∞_, we sample configurations in a trajectory in 0.5 ps increments to reduce temporal correlations. Statistical uncertainties for both *C*_V_ and *D*_∞_ were estimated by block averaging. The self-diffusion coefficient at the infinite-system limit *D*_∞_ was computed from the MD trajectories by first computing the finite-size diffusivity *D*. We compute *D* directly from our MD trajectories from the linear slope of the MSD relative to time *t* using MSD(*t*) ≈ 6*Dt* + *C*, where the slope was obtained by identifying the diffusive regime (*t* ≥ 100 ps) where there is a linear relationship between the MSD and time.

We then apply the Yeh–Hummer correction^80^ to get the infinite-limit diffusion coefficient *D*_∞_3
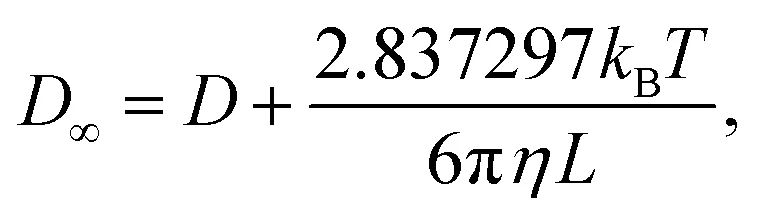
where *D* is the diffusion coefficient obtained from MD simulations with periodic simulation cells, and *D*_∞_ is the diffusion coefficient in the infinite-system limit with Boltzmann constant *k*_B_, temperature *T*, length of simulation box *L* and *η* the shear viscosity (here taken as the experimental value 8.88 × 10^−4^ Pa s at 298 K).^104^

## Author contributions

N. H. D. acquired, investigated, analyzed MD results. F. S. computed DFT data. X. W., and D. K. validated and analyzed MD results. N. H. D. and X. W. developed data analysis code. N. H. D. prepared all figures. N. H. D., D. S. K., and B. C. wrote the manuscript. F. S., G. B., J. H. and B. C. acquired funding. G. B., J. H. and B. C. conceptualized the project. B. C. designed the project. D. S. K. and B. C. supervised the project. All authors revised and approved the manuscript.

## Conflicts of interest

B. C. has an equity stake in AIMATX Inc. University of California, Berkeley has filed an non-provisional patent for the Latent Ewald Summation algorithm.

## Supplementary Material

DD-005-D6DD00571C-s001

## Data Availability

The CP2K input files, training set, training and analysis scripts, and trained CACE potentials are available at https://github.com/namdao2404/water-benchmark. The dataset of DFT calculations on snapshots of water are available at https://zenodo.org/records/17509496, including the training and validation data for all 29 functionals considered in this work. The Zenodo repository contains the complete DFT datasets used to train and validate models, while the GitHub repository contains the corresponding input files, scripts, and trained potentials needed to reproduce the calculations and analysis. Code availability: the CACE package is publicly available at https://github.com/BingqingCheng/cace. The LES package is available from the same repository and implemented as a Ewald module. Further implementation details on BEC calculations are provided at https://github.com/BingqingCheng/LES-BEC. Supplementary information is (SI) available. See DOI: https://doi.org/10.1039/d6dd00571c.
