# Supplementary material for: Systematic trends in water properties across Jacob's ladder density functionals
Source: Digit Discov. 2026 Sep 25;5(11):4989–5000. doi: 10.1039/d6dd00571c (PMC13636407; doi:10.1039/d6dd00571c)
Supplement: Supplementary file 2 [file DD-005-D6DD00571C-s001.pdf]

Supplementary Information (SI) for Digital Discovery.
This journal is © The Royal Society of Chemistry 2026
Supplementary Information (SI) for Digital Discovery.
This journal is © The Royal Society of Chemistry

Supplementary Information

Systematic Trends in Water Properties Across Jacob's Ladder Density Functionals

Nam H. Đào,[1] Daniel S. King,[2,*] Frederick Stein,[3,4,†] Xiaoyu Wang,[1] Dongjin Kim,[1] Gerit Brandenburg,[3] Jürg Hutter,[3] and Bingqing Cheng[1,2,5,‡]

[1] *Department of Chemistry, UC Berkeley, California 94720, United States*

[2] *Bakar Institute of Digital Materials for the Planet, UC Berkeley, California 94720, United States*

[3] *Department of Chemistry, University of Zurich, Winterthurerstrasse 190, 8057 Zurich, Switzerland*

[4] *Center for Advanced Systems Understanding (CASUS), Helmholtz-Zentrum Dresden-Rossendorf e.V. (HZDR), Bautzner Landstraße 400, 01328 Dresden, Germany*

[5] *The Institute of Science and Technology Austria, Am Campus 1, 3400 Klosterneuburg, Austria*

(Dated: August 3, 2026)

* king1305@berkeley.edu

† f.stein@hzdr.de

‡ bingqingcheng@berkeley.edu

CONTENTS

I. MLIP Training 3

II. Model Extrapolation Test 5

III. TRPMD Simulations 7

Simulation details 7

Impact of Nuclear quantum effects on the oxygen–oxygen radial distribution function 7

Impact of Nuclear quantum effects on the infrared spectrum of bulk water 8

IV. Hexagonal Ice IR spectra 10

V. Radial Distribution Function Comparison 12

VI. Computing Heat Capacity and Diffusion 13

Heat Capacity 13

Self-Diffusion Coefficient 14

FFT-Based MSD Calculation 14

Block Averaging and Uncertainty Estimation 15

VII. MLIP MD vs. MLIP MD comparison 17

VIII. MLIP MD vs. AIMD comparison 18

IX. Finite Size Convergence Test 21

Radial Distribution Function 21

Heat Capacity C_V 21

Diffusion Coefficient D_∞ 22

X. Correlation Studies 24

References 26

I. MLIP TRAINING

Below we plot a comparison of short-range (SR) CACE-SR vs. long-range (LR) CACE-LR force root mean squared error (RMSE). For most density funcioonal approximations (DFAs), CACE-LR shows slight improvement or equal performance to CACE-SR models trained on the energy and forces of a given functional.

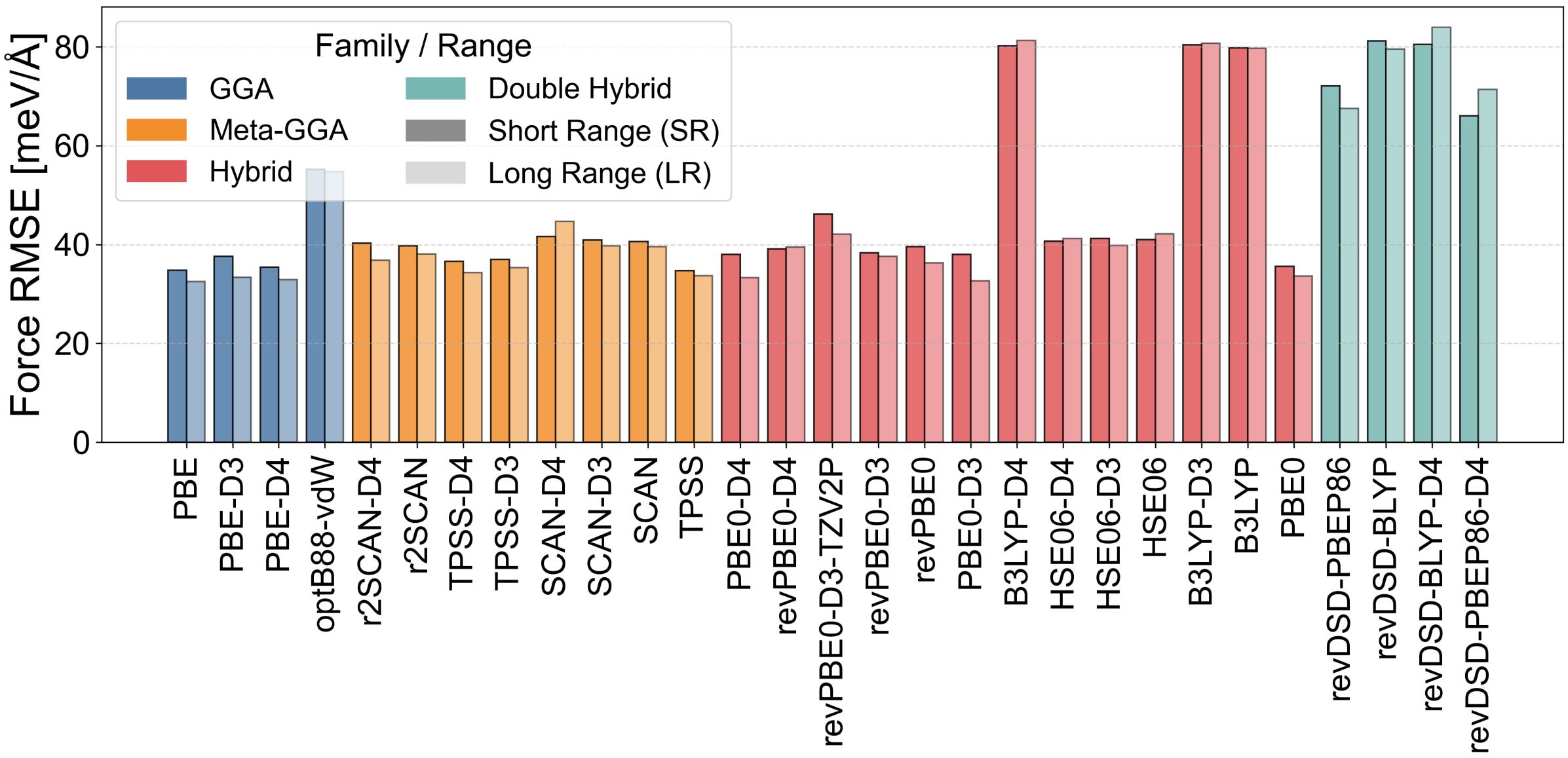

Figure S1. A comparison of force RMSE in meV/Å. Functionals are colored by family, each with a left bar (darker shade) representing CACE-SR RMSE and a right bar (lighter shade) denoting the CACE-SR RMSE.

Below we plot a comparison of the energy RMSE of all CACE-LR models.

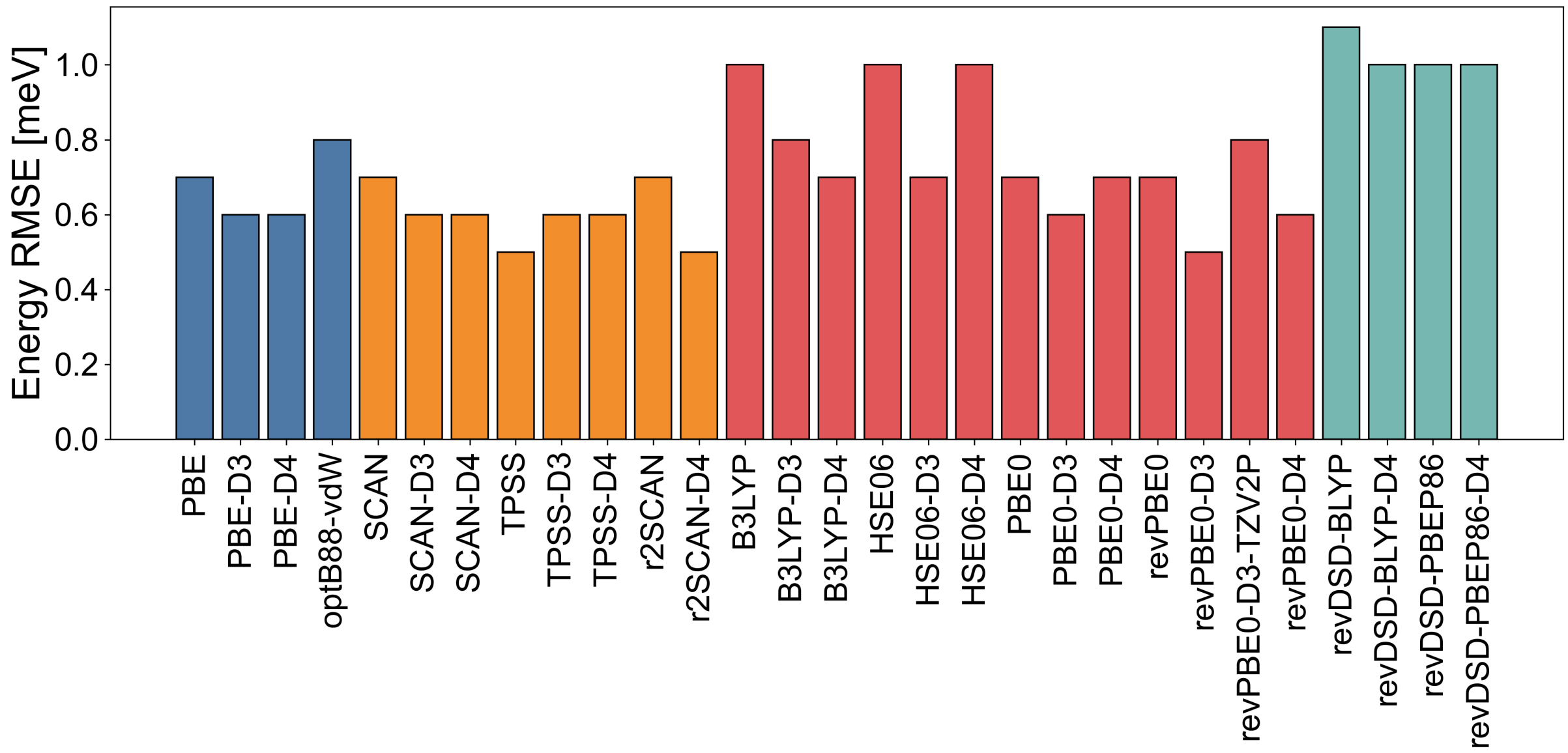

Figure S2. A comparison of energy RMSE errors in meV.

II. MODEL EXTRAPOLATION TEST

To test for model extrapolation during molecular dynamics simulations, we recomputed DFT single-point calculations on 100 uncorrelated snapshots from 1 ns trajectories of PBE and revPBE0 at 300 K. Fig. S3 and Fig. S4 compare the CACE-LR predictions against the recomputed DFT reference for both functionals. The in-trajectory energy RMSEs are 0.18 meV/atom for both PBE and revPBE0-D3, and the force RMSEs are 15.13 and 18.90 meV/Å, respectively. These values are comparable to the validation errors reported in Fig. S2 and Fig. 1 of the main manuscript, demonstrating that the CACE-LR models remain faithful to their target DFA potential energy surface on their trajectories.

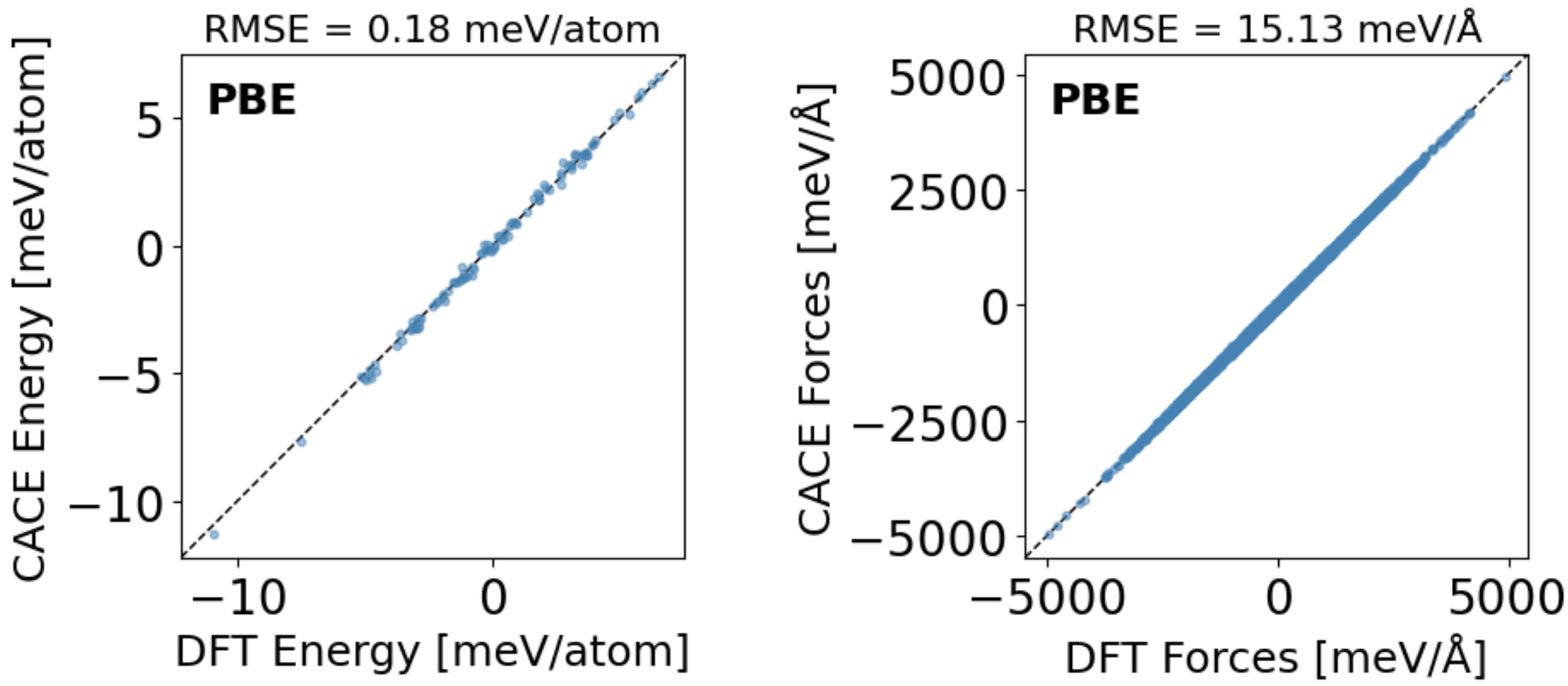

Figure S3. In-trajectory validation of the CACE-LR surrogate against target DFT for the PBE functional. Predicted energies (left, meV/atom) and forces (right, meV/Å) from the CACE-LR model are plotted against DFT single-point recomputations for 100 uncorrelated snapshots drawn from the 1 ns classical MD trajectory at 300 K. Energy RMSE = 0.18 meV/atom; force RMSE = 15.13 meV/Å.

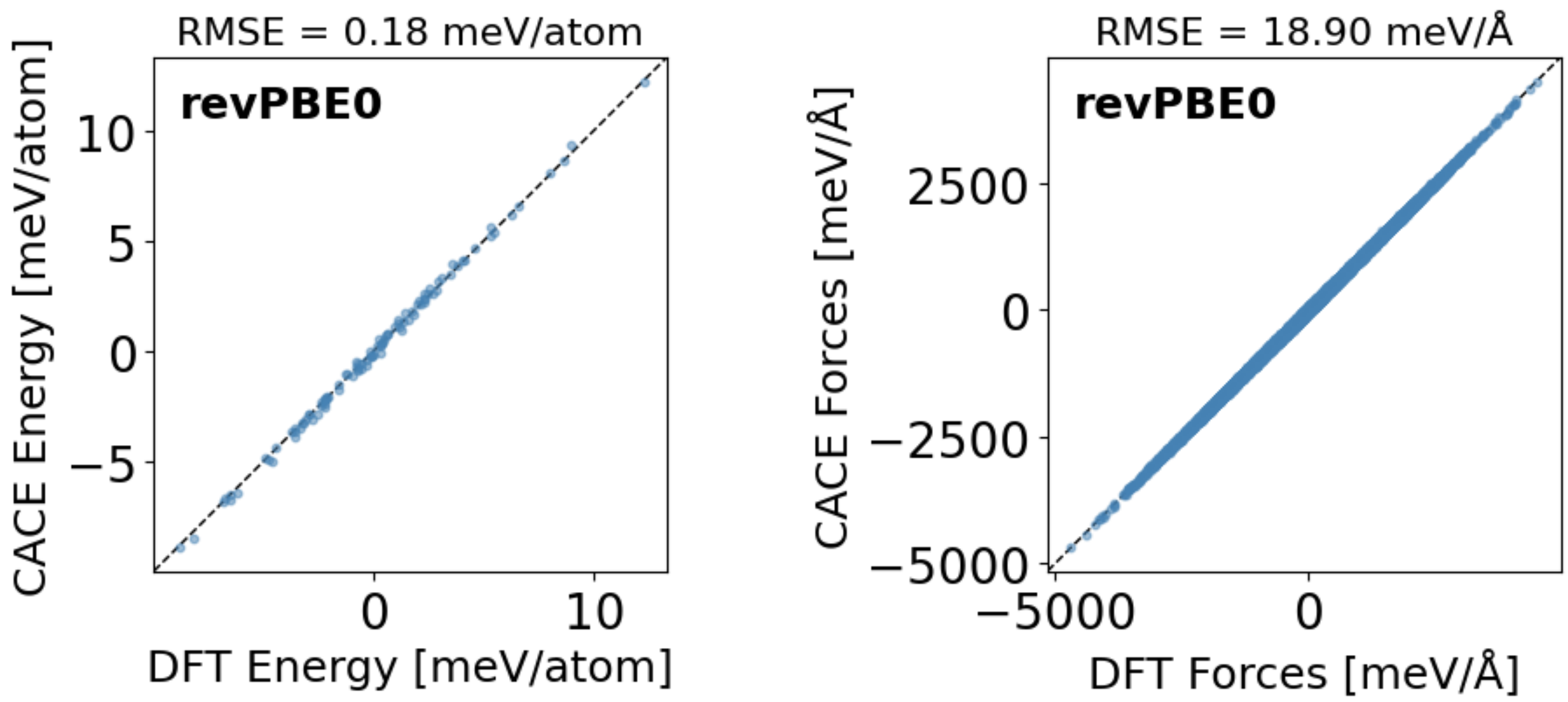

Figure S4. In-trajectory validation of the CACE-LR surrogate against target DFT for the revPBE0-D3 functional. Predicted energies (left, meV/atom) and forces (right, meV/Å) from the CACE-LR model are plotted against DFT single-point recomputations for 100 uncorrelated snapshots drawn from the 1 ns classical MD trajectory at 300 K. Energy RMSE = 0.18 meV/atom; force RMSE = 18.90 meV/Å.

III. TRPMD SIMULATIONS

To provide direct internal validation of the classical-nuclei approximation used throughout the main manuscript, we performed thermostatted ring-polymer molecular dynamics (TRPMD) simulations for five representative functionals spanning the four rungs of Jacob's ladder: PBE (GGA), SCAN (meta-GGA), B3LYP (hybrid), revPBE0-D3 (hybrid), and revDSD-PBEP86 (double hybrid).

Simulation details

Simulations were carried out in the NVT ensemble at $T = 300$ K for 100 ps with 25 ps of equilibration time on the same $1 \times 1 \times 1$ cubic box of 64 H_2O molecules ($L = 12.42$ Å, $\rho = 997$ kg/m^3) used in the classical MD runs. The ring polymer was discretised into 32 beads carrying physical atomic masses.

Nuclear equations of motion were integrated with a timestep of $\Delta t = 0.5$ fs using the path-integral Langevin equation (PILE-L) thermostat [1], with the internal-mode thermostat friction parameter $\lambda = 0.5$, following Rossi, Ceriotti, and Manolopoulos [2].

Structural observables (gOO(r)) are evaluated from single-bead (bead-0) positions, while vibrational observables (IR spectra) are evaluated from the ring-polymer centroid trajectory. Bead-0 positions and centroid positions and velocities were dumped every step (0.5 fs) into dedicated trajectory files, enabling frame-for-frame post-processing with the same analysis pipeline used for the classical trajectories.

Thermodynamic observables that require the correct quantum kinetic-energy estimator (e.g., C_V) cannot be recovered from bead-0 quantities alone and are therefore not reported from the TRPMD runs; the classical values with Levitt [3] corrections applied discussed in the main manuscript remain our primary C_V estimates.

Impact of Nuclear quantum effects on the oxygen–oxygen radial distribution function

Fig. S5 compares the oxygen–oxygen radial distribution functions $g_{\mathrm{OO}}(r)$ computed from classical MD and from TRPMD for each of the five representative functionals, alongside

the experimental reference of Soper [4]. The magnitude of the NQE-induced modification is functional-dependent and generally decreases toward the higher rungs of Jacon's Ladder: PBE, which most severely overstructures liquid water during classical simulation, shows the largest quantum softening (first-peak reduction from 3.79 to 3.30, together with a modest reduction of the second peak near 4.4 Å), while the higher-rung hybrid and double-hybrid functionals exhibit only minor differences between classical and TRPMD $g_{OO}(r)$. For revDSD-PBEP86, the classical and TRPMD oxygen–oxygen radial distribution functions are essentially indistinguishable, both agreeing closely with the experimental reference.

Crucially, in all five cases the NQE-induced modification of $g_{OO}(r)$ is smaller than the DFA-to-DFA spread observed in the classical benchmark, and the functional-accuracy ranking against the experimental reference is preserved with the inclusion of NQEs.

These results provide direct confirmation of the well-documented observation that NQEs have a modest and largely non-ranking-altering effect on the oxygen–oxygen structure of bulk liquid water [5, 6], and support our use of experimental $g_{OO}(r)$ as a legitimate reference for classical-nuclei benchmarking of the DFAs in the main manuscript.

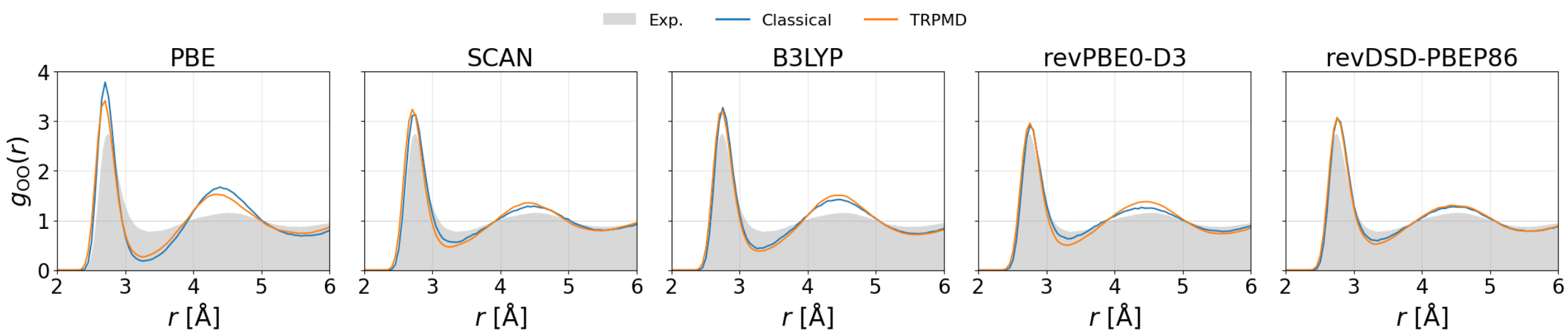

Figure S5. Oxygen–oxygen radial distribution functions $g_{OO}(r)$ of liquid water at 300 K for the five representative functionals spanning Jacob's ladder computed by classical MD and TRPOMD. Both are compared to the experimental reference of Soper [4].

Impact of Nuclear quantum effects on the infrared spectrum of bulk water

Fig. S6 compares the NQE-shifted classical and TRPMD IR spectra for the five representative functionals. Across all five, the band positions of the NQE-shifted classical spectra track the corresponding TRPMD peaks closely, with only minor deviations in the librational region.

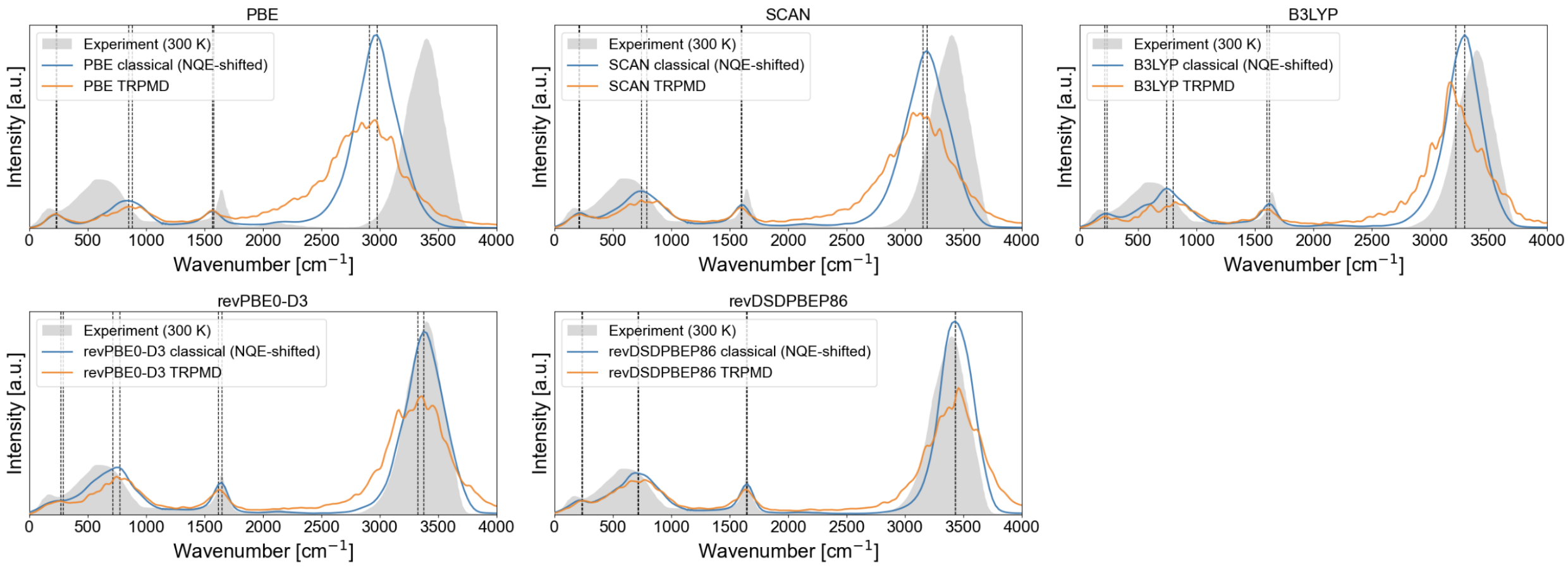

Figure S6. Infrared spectra of liquid water at 300 K for the five representative functionals (PBE, SCAN, B3LYP, revPBE0-D3, revDSDPBEP86 spanning Jacob's ladder computed by empirically shifted classical MD and TRPMD. Both are compared to the experimental reference. [7, 8]

IV. HEXAGONAL ICE IR SPECTRA

The hexagonal ice ih IR spectra at 100 K was also computed for this study along with the bulk water IR spectra discussed in the main manuscript. The IR spectra peaks were manually shifted to account for nuclear quantum effects. Details on values used are provided in the Methods section of the main manuscript.

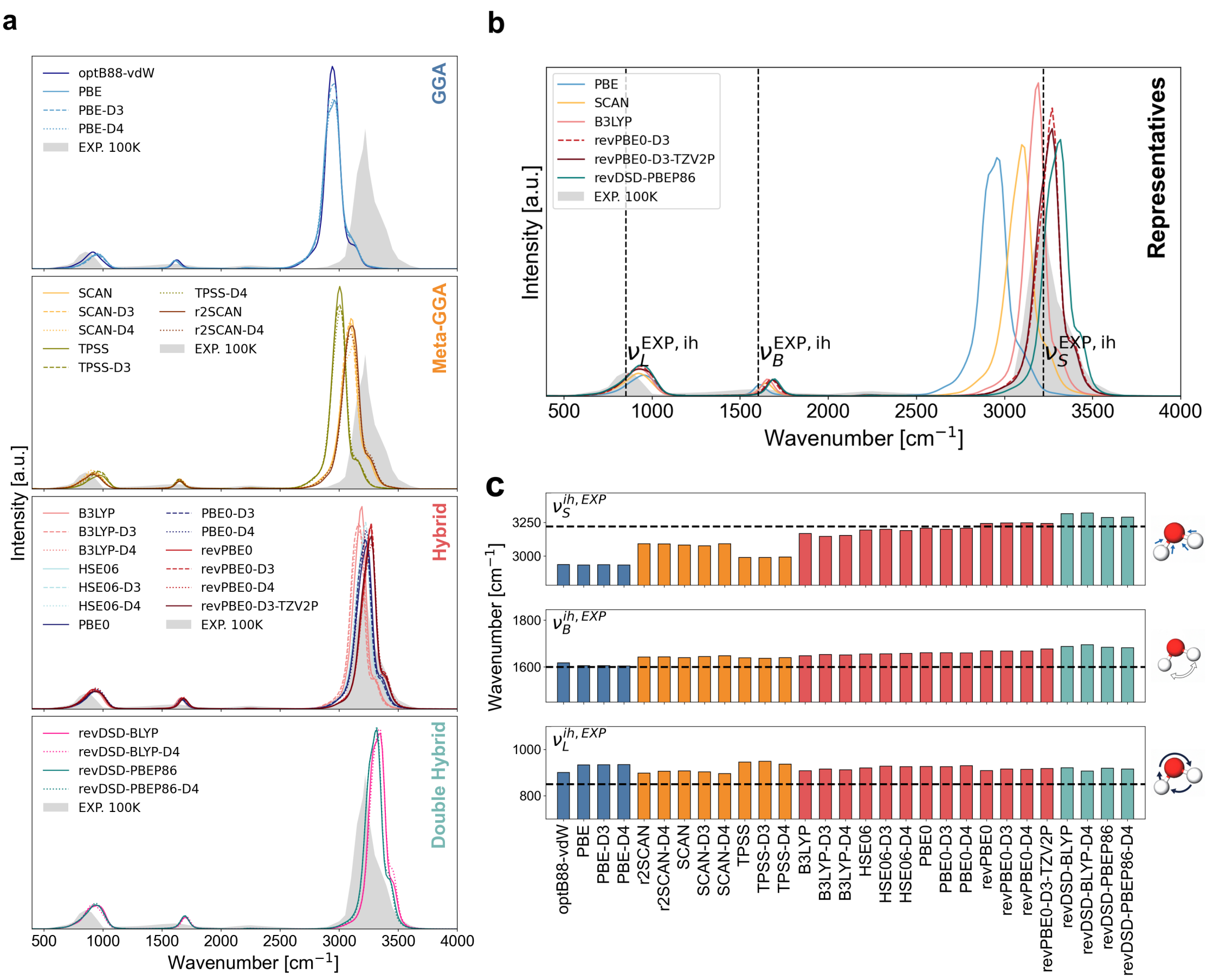

Figure S7. Infrared spectra of hexagonal ice Ih at 100 K computed by density functional approximations using Born effective charge (BEC) tensors from long-range models. The experimental infrared spectra are provided for reference [9]. **a** Infrared spectra of hexagonal ice for all functionals separated by rung on Jacob's Ladder. **b** Infrared spectra of hexagonal ice for select functionals with experimental peaks shown with dashed lines. **c** Comparison of all density functional vibrational modes ν_T^{ih}, ν_L^{ih}, ν_B^{ih}, and ν_S^{ih} compared to the experimental values.

The infrared spectrum of hexagonal ice complements that of liquid water, as spectral shifts and band shapes reflect changes in hydrogen-bond ordering and dipole correlations. As for

bulk water, the ice Ih IR spectra at 100 K were post-processed with literature-motivated empirical corrections to emulate nuclear quantum effects [10, 11]. Specific values are provided in the Methods section. The computed IR spectra for all the DFAs successfully recover the key ice vs liquid contrasts observed experimentally and in first-principles studies: sharper librations, a narrower bending band, and a red-shifted, tighter OH stretch envelope for ice compared to liquid water [12]. Quantitatively, the post-NQE maxima from Fig. S7 fall in the established ice Ih windows. The hierarchy in error of vibrational modes in ice reproduces the liquid-phase trend: adding exact exchange and perturbative correlation improves intramolecular frequencies while collective librations remain challenging, reflecting incomplete softening in the ordered H-bond network.

V. RADIAL DISTRIBUTION FUNCTION COMPARISON

Below we compare the peak radii r_1, r_2, and heights $g_{OO}(r_1)$, $g_{OO}(r_2)$ of the first and second peaks of the oxygen–oxygen radial distribution function $g_{OO}(r)$.

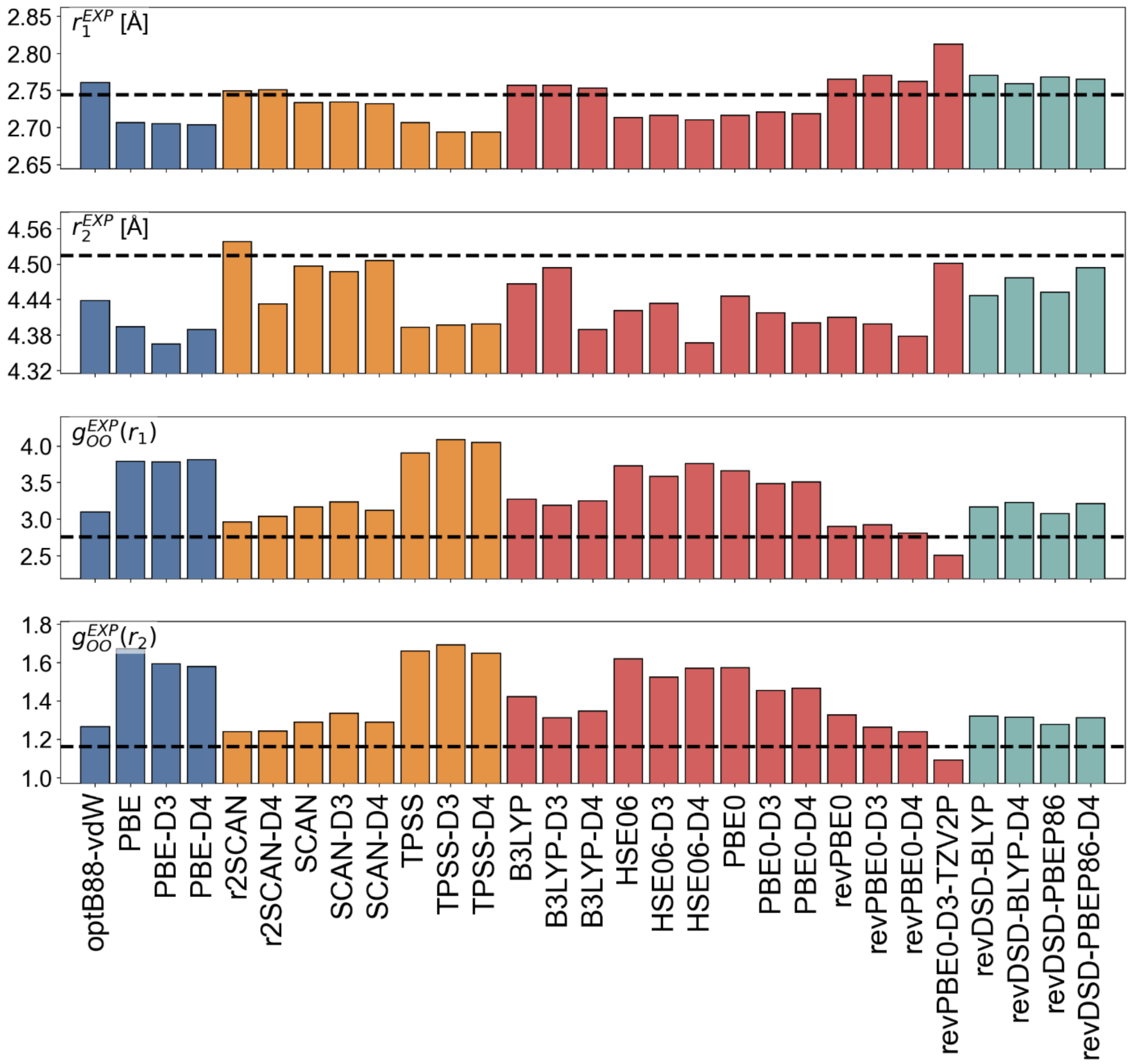

Figure S8. Comparison of r_1, r_2, $g_{OO}(r_1)$ and $g_{OO}(r_2)$.

VI. COMPUTING HEAT CAPACITY AND DIFFUSION

Configurations used to compute the heat capacity C_V and the diffusion coefficient D_∞ were both sampled every 0.5 ps from 1 ns NVT trajectories, but with different equilibration cutoffs reflecting the different convergence timescales of the two observables. For C_V, the first 250 ps were discarded as equilibration, yielding 750 ps of production data per functional; this longer cutoff is required because the total potential energy exhibits slow drift over the first ∼200 ps for several functionals (most notably HSE06, PBE, TPSS-D4, and PBE0).

For D_∞, only the first 50 ps were discarded, yielding 950 ps of production data per functional, as the oxygen MSD enters the diffusive regime well within this window for all functionals studied.

Uncertainty for both quantities was estimated by block averaging. A global choice of $M = 5$ non-overlapping, contiguous blocks was applied uniformly across all functionals for both C_V and D_∞, set by inspection of the integrated autocorrelation time of the total potential energy across functionals.

Heat Capacity

The molar heat capacity C_V is computed from the fluctuation formula

$$C_V = \frac{N_A}{N\, k_B T^2}\, \mathrm{Var}(E), \tag{1}$$

where N is the number of water molecules in the simulation cell, k_B is the Boltzmann constant, T is the temperature of the production trajectory, and N_A is Avogadro's number. The variance of the total potential energy is estimated using the unbiased sample variance over the full production trajectory:

$$\mathrm{Var}(E) = \frac{1}{n-1} \sum_{j=1}^{n} \left(E_j - \bar{E}\right)^2, \tag{2}$$

where n is the number of sampled configurations and $\bar{E}$ is the mean total energy. The reported C_V is obtained by inserting this full-trajectory variance into the fluctuation formula.

To estimate the statistical uncertainty, the production trajectory is divided into $M = 5$ non-overlapping, contiguous blocks of equal size $B = n/M$. For each block i, a block heat

capacity is computed independently using the block-local variance:

$$C_{V,i} = \frac{N_A}{N\,k_B T^2}\,\mathrm{Var}_i(E), \tag{3}$$

where $\mathrm{Var}_i(E)$ is the unbiased sample variance computed using only configurations within block i. The statistical uncertainty is reported as the standard error of the mean of the block values:

$$\sigma_{C_V} = \frac{1}{\sqrt{M}}\sqrt{\frac{1}{M-1}\sum_{i=1}^{M}\left(C_{V,i}-\bar{C}_V\right)^2}, \qquad \bar{C}_V = \frac{1}{M}\sum_{i=1}^{M} C_{V,i}. \tag{4}$$

The final reported value is therefore

$$C_V \pm \sigma_{C_V},$$

where C_V is the full-trajectory estimate and σ_{C_V} is the block SEM.

Self-Diffusion Coefficient

The self-diffusion coefficient D is computed from the mean squared displacement (MSD) of oxygen atoms via the Einstein relation:

$$D = \lim_{t\to\infty}\frac{1}{6}\frac{d}{dt}\left\langle |\mathbf{r}(t)-\mathbf{r}(0)|^2\right\rangle. \tag{5}$$

In practice, D is obtained from a linear regression of the MSD in the diffusive regime:

$$\mathrm{MSD}(t) = \left\langle |\mathbf{r}(t)-\mathbf{r}(0)|^2\right\rangle = 6Dt + b, \tag{6}$$

so that

$$D = \frac{\text{slope}}{6}. \tag{7}$$

FFT-Based MSD Calculation

For a trajectory containing N frames, the mean squared displacement is evaluated as a function of the discrete time lag m,

$$m = 0, 1, \ldots, N-1, \tag{8}$$

where m is a dimensionless frame index corresponding to physical time $t_m = m\,\Delta t$, with Δt the effective time spacing between analyzed frames.

The MSD at lag m is computed using the FFT-based algorithm of Calandrini (also referred to as the Borodin method) [13]:

$$\mathrm{MSD}(m) = \frac{S_1(m) - 2S_2(m)}{N-m}, \tag{9}$$

where $S_1(m)$ contains squared-position terms and $S_2(m)$ is the position autocorrelation function evaluated via the convolution theorem using fast Fourier transforms (FFT). This approach reduces the computational scaling from $\mathcal{O}(N^2)$ to $\mathcal{O}(N \log N)$.

Block Averaging and Uncertainty Estimation

After discarding the first 50 ps as equilibration, the remaining 950 ps production trajectory is divided into $M = 5$ non-overlapping blocks of equal length (190 ps each). For each block i, an independent MSD is computed and a diffusion coefficient D_i is extracted via linear regression in the diffusive regime ($t \geq 100$ ps):

$$D_i = \frac{1}{6}\,\mathrm{slope}_i, \tag{10}$$

where slope_i is the fitted slope of $\mathrm{MSD}_i(t)$. The mean diffusion coefficient is

$$\bar{D} = \frac{1}{M}\sum_{i=1}^{M} D_i. \tag{11}$$

The block-to-block sample standard deviation (using $M-1$ in the denominator) is

$$s = \sqrt{\frac{1}{M-1}\sum_{i=1}^{M}\left(D_i - \bar{D}\right)^2}. \tag{12}$$

The statistical uncertainty associated with finite sampling (standard error of the mean, SEM) is

$$\sigma = \frac{s}{\sqrt{M}}. \tag{13}$$

The final reported value is therefore

$$D = \bar{D} \pm \sigma. \tag{14}$$

Below we show a demonstrative example of our calculation of the self-diffusion coefficient. The diffusive regime is identified by applying a fixed time cutoff of $t \geq 100$ ps, beyond which the MSD exhibits a clear linear dependence on time consistent with diffusion for all DFAs. We then extract the diffusion coefficient from the slope of this linear region using the Einstein relation discussed in the Methods section of the main paper.

For the TPSS, TPSS-D3, and TPSS-D4 functionals, the water dynamics are sufficiently slow that a single 1 ns trajectory does not reach the diffusive regime within the fit window, consistent with their strongly over-structured radial distribution functions (Fig. 3 from the main manuscript). To obtain converged D_∞ estimates for these three DFAs, two additional independent 1 ns replicates were run per functional, and the diffusion coefficient was extracted from the concatenated MSD across all three replicates using the same block-averaging protocol described above.

VII. MLIP MD VS. MLIP MD COMPARISON

To verify consistency of our MLIP MD results with prior literature that uses the same CACE architecture and MD configurations [14], we evaluate the similarity in oxygen–oxygen radial distribution function peak positions r_1, r_2 and heights $g_{OO}(r_1)$, $g_{OO}(r_2)$ for bulk water at 300 K. We also compare the diffusion coefficient D_∞ between the two works.

Functional	MLIP r_1	MLIP r_2	MLIP $g_{OO}(r_1)$	MLIP $g_{OO}(r_2)$	MLIP D_∞
revPBE0-D3	2.77	4.40	2.92	1.26	0.13
revPBE0-D3-TZV2P	2.81	4.50	2.51	1.09	0.30
revPBE0-D3-TZV2P (from Cheng et al. [14])	~ 2.8	~ 4.5	~ 2.6	~ 1.1	0.26
REF. [4, 15]	2.74 [4]	4.52 [4]	2.76 [4]	1.16 [4]	0.28 [15]

Table S1. Comparison of structural parameters and self-diffusion coefficients with Ref. [14].

We see that there is close agreement between the two sets of results, as expected from training on the same dataset.

VIII. MLIP MD VS. AIMD COMPARISON

We also further compare the results of MLIP-driven MD in this work with prior AIMD literature on the structural, response, and thermodynamic properties of bulk water for various functionals [16]. As Tab. S2 shows, there is strong agreement between the two sets of results for the peak positions r_1 but some over-structuring by the MLIPs employed in this work of the peak heights $g_{OO}(r_1)$. In contrast, revPBE0-D3-TZV2P closely matches the previous AIMD results. There is also general agreement for the infinite-limit diffusion coefficients D_∞.

Functional	MLIP r_1	MLIP $g_{OO}(r_1)$	MLIP D_∞	AIMD r_1	AIMD $g_{OO}(r_1)$	AIMD D_∞
optB88-vdW	2.76	3.10	0.12	2.74	2.92	0.14
PBE	2.71	3.79	0.06	2.72	3.28	—
PBE-D3	2.71	3.79	0.06	2.73	3.05	—
SCAN	2.73	3.17	0.10	2.76	3.25	0.08
TPSS	2.71	3.90	0.06	2.69	3.70	—
B3LYP	2.76	3.27	0.08	2.77	2.78	—
PBE0	2.72	3.66	0.06	2.71	2.97	0.12
PBE0-D3	2.72	3.48	0.08	2.74	2.86	—
revPBE0-D3	2.77	2.92	0.13	2.80	2.58	0.27
revPBE0-D3-TZV2P	2.81	2.51	0.30	2.80	2.58	0.27
REF. [4, 15]	—	—	—	2.74 [4]	2.76 [4]	0.28 [15]

Table S2. Comparison of the self-diffusion coefficient as well as the bulk water oxygen–oxygen radial distribution function first peak position r_1 in Å and height $g_{OO}(r_1)$ using MLIP (this work) and AIMD (from Villard et. al [16]) values for selected functionals. Experimental radial distribution peaks [4] and the MB-Pol classical reference diffusion coefficient [15] are shown for reference.

We provide below a bar plot of the above table for ease of comparison.

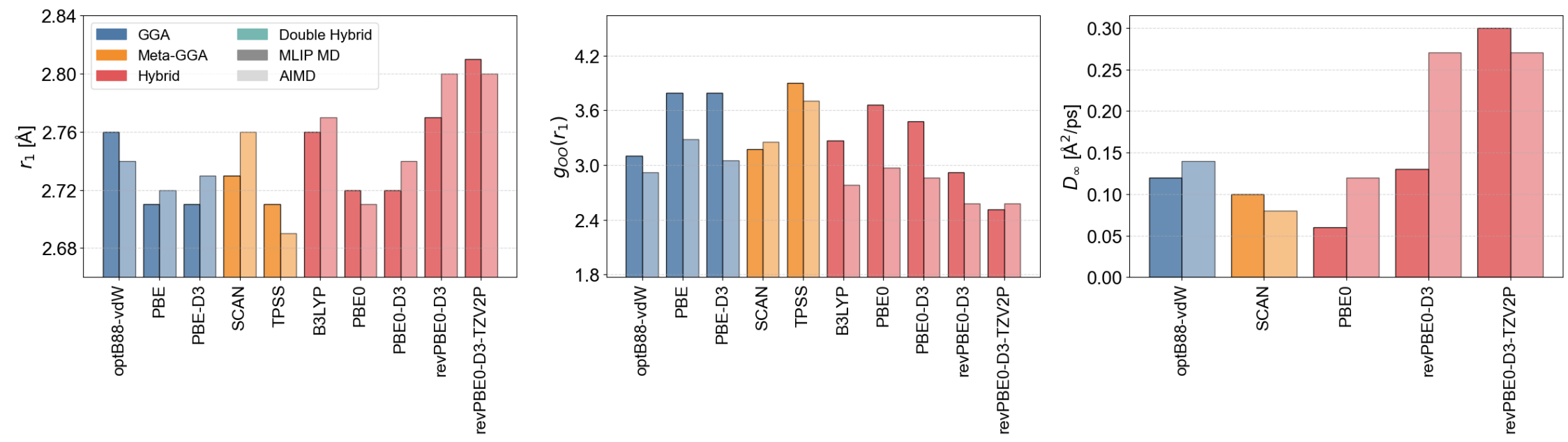

Figure S9. Comparison of r_1, $g_{OO}(r_1)$ and D_∞ AIMD vs MLIP generated values.

Table S3. Comparison of vibrational band frequencies of water from MLIP MD and AIMD simulations. The experimental IR spectra are taken from Bertie and Lan [7, 8] and replotted to find peaks for each vibrational mode. AIMD reference values computed using the revPBE0-D3 function with the TZV2P basis set extracted from Marsalek et al. [5] are provided for comparison. Values are reported in cm^{-1}.

**Functional**	**MLIP MD (this work) at 300 K**				**AIMD at 300 K**			
	$\nu_T^{classical}$	$\nu_L^{classical}$	$\nu_B^{classical}$	$\nu_S^{classical}$	$\nu_T^{classical}$	$\nu_L^{classical}$	$\nu_B^{classical}$	$\nu_S^{classical}$
revPBE0-D3 [5]	193.1	720.8	1708.5	3550.5	$\sim$ 150	$\sim$ 670	$\sim$ 1680	$\sim$ 3600
revPBE0-D3-TZV2P [5]	141.8	667.4	1681.8	3630.6	$\sim$ 150	$\sim$ 670	$\sim$ 1680	$\sim$ 3600
EXP. [7, 8]	–	–	–	–	178.2	620.0	1652.5	3380.0

We also compare the molar heat capacity computed using PBE as computed by MLIP MD in this work and AIMD PBE predictions from Pascal et al. [17]. The MLIP MD simulations performed at 300 K using a 64-molecule system for the PBE functional in this work resulted in a heat capacity C_V of 120.0, representing a overestimation relative to the AIMD value of 89.3 computed by Pascal et al. [17] and slightly overestimating the experimental heat capacity 74.5 $J/(mol \cdot K)$. As discussed in the main manuscript, we attribute this discrepancy to the difference in sampling time between the two protocols.

Finally, we include the IR spectra peaks computed by prior AIMD literature for the functional revPBE0-D3 [5, 18]. We compare the results of our MLIP-MD IR spectrum with corresponding AIMD works for the peaks of the vibrational modes ν_T, ν_L, ν_B, and ν_S. These modes correspond to the translational, librational, bending, and stretching bands, respec-

tively. Values computed from running MD and directly reported without any corrections for nuclear quantum effects are denoted by the superscript “classical”.

IX. FINITE SIZE CONVERGENCE TEST

To assess whether the 64-molecule simulation cell used throughout the main manuscript is sufficient to converge the properties of interest, we performed a systematic finite-size analysis for 5 representative density functionals approximations considered in this study. For each functional, a second set of 2×2×2 cell (containing 512 molecules) classical MD trajectories were generated with the corresponding CACE-LR MLIPs.

Both simulations were run for 1 ns in the NVT ensemble at 300 K with 50 ps of equilibration time and the experimental density, using identical integrator settings, thermostat parameters, and analysis protocols.

We focus on the convergence of radial distribution functions as well as dynamical (D_∞ and thermodynamic (C_V) properties.

Radial Distribution Function

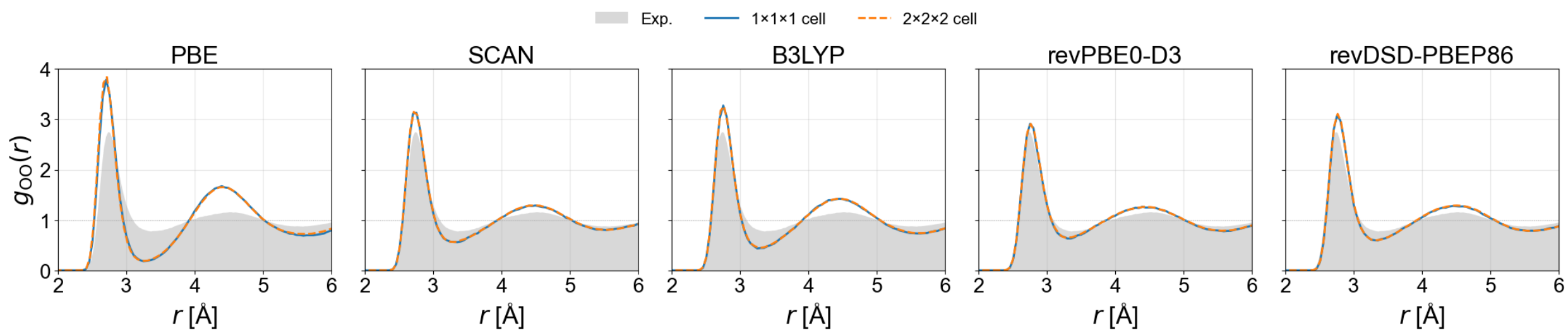

Figure S10. Finite-size convergence of the oxygen–oxygen radial distribution function $g_{OO}(r)$ for the five representative functionals spanning Jacob's ladder. Solid orange lines show results from the 1×1×1 simulation cell, and dashed black lines show results from the 2×2×2 cell. Both were obtained from 1 ns classical MD trajectories at 300 K and the experimental density using the corresponding CACE-LR MLIP. The experimental references from Soper [4].

Fig. S10 shows that increasing the system size from 64 to 512 molecules has little effect on the oxygen–oxygen radial distribution functions for all five functionals, demonstrating that the 64-molecule cell is sufficient for converged structural analysis.

Heat Capacity C_V

Heat capacity analysis was performed on both the 1×1×1 and 2×2×2 trajectories using the block-averaging protocol described above. As shown in Fig. S11, the results are converged with respect to system size across all five representative functionals.

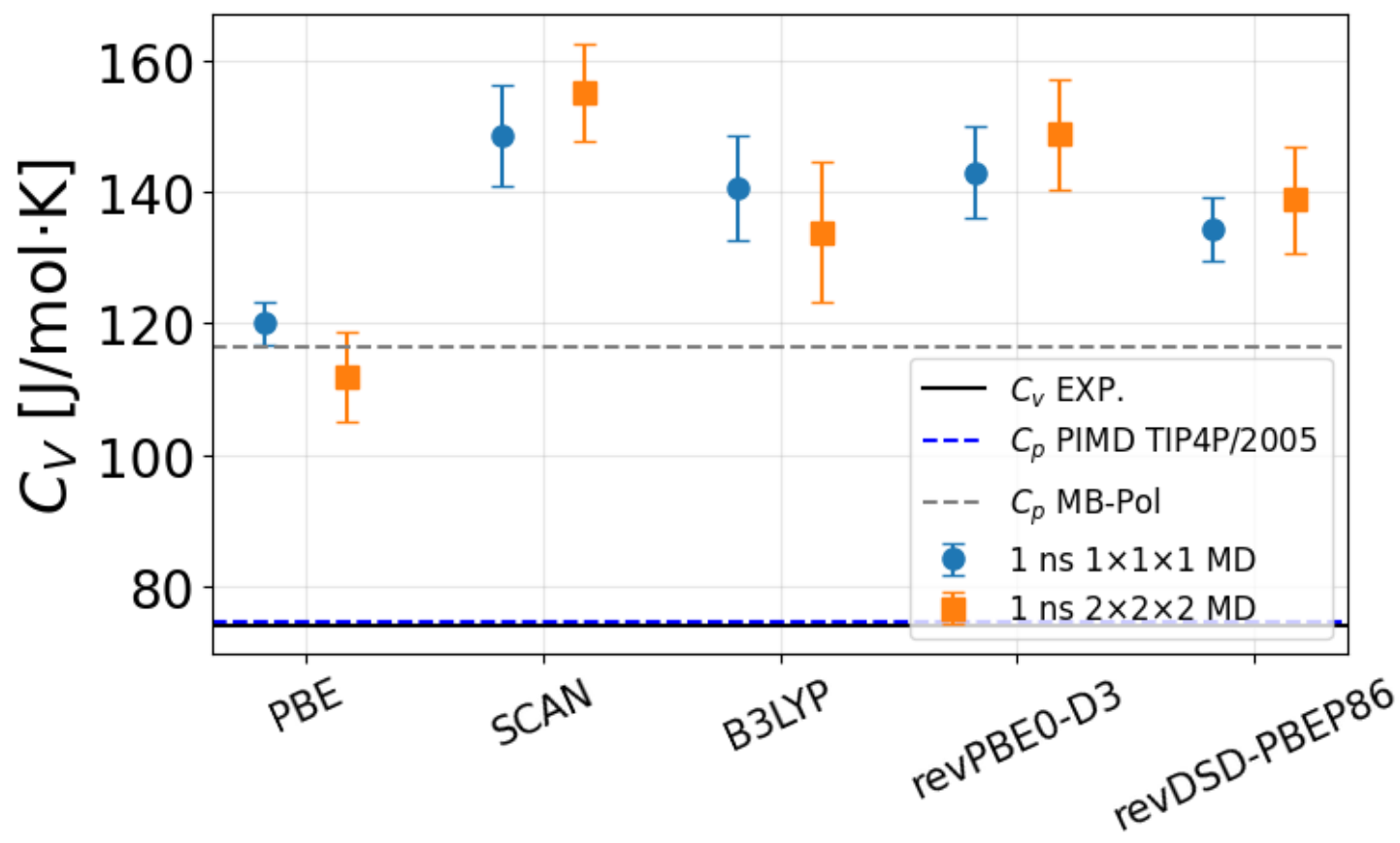

Figure S11. Finite-size convergence of the Levitt-corrected molar heat capacity C_V for the five representative functionals spanning Jacob's ladder. black circles show results from the 1×1×1 simulation cell (64 H_2O), and orange squares show results from the 2×2×2 cell (512 H_2O). Both were obtained from 1 ns classical MD trajectories at 300 K and the experimental density using the corresponding CACE-LR MLIP. Error bars are 1σ SEMs from $M = 5$ block averaging. The two box sizes agree within combined uncertainty for all functionals

Diffusion Coefficient D_∞

Finite size convergence was similarly computed for the Yeh–Hummer-corrected [19] diffusion coefficient D_∞. As shown in Fig. S12, the two box sizes agree closely with some slight discrepancy for PBE and SCAN; we attribute these primarily to the use of the experimental shear viscosity in the Yeh–Hummer correction, which is a common approximation but not strictly self-consistent with the functional-specific dynamics. A functional-specific viscosity η, which is inaccessible from the 1 ns trajectories used here due to insufficient Green–Kubo plateau convergence, would be expected to reduce these residual discrepancies.

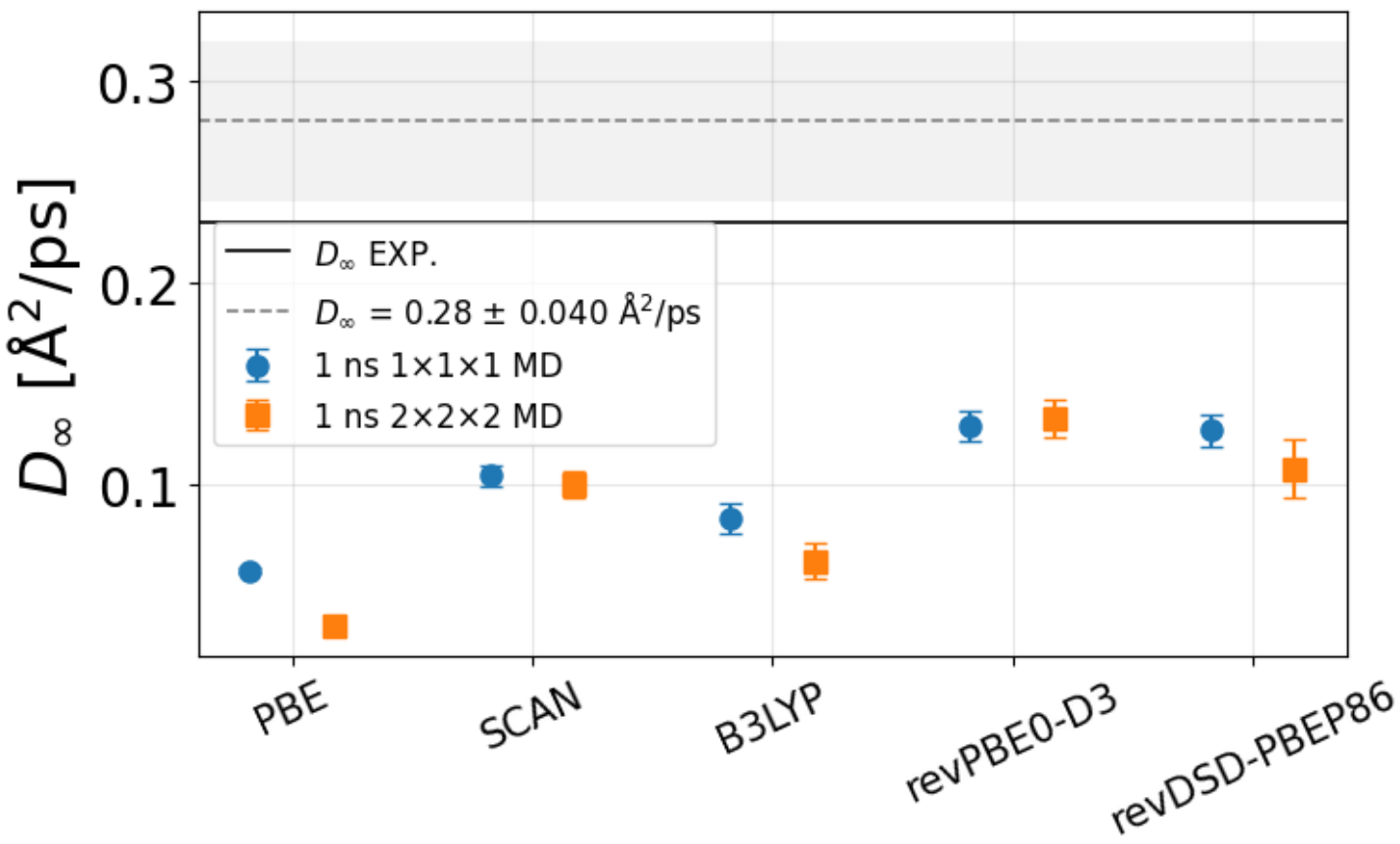

Figure S12. Finite-size convergence of the self-diffusion coefficient D_∞ for the five representative functionals spanning Jacob's ladder. black circles show results from the 1×1×1 simulation cell (64 H_2O), and orange squares show results from the 2×2×2 cell (512 H_2O). The Yeh–Hummer finite-size correction has been applied to both, using the experimental shear viscosity $\eta = 8.88 \times 10^{-4}$ Pa·s [20]. Error bars are 1σ SEMs from $M = 5$ block averaging. The experimental (0.229 Å^2/ps) [21] value and MB-pol value [15] are included as reference.

X. CORRELATION STUDIES

Below we compute the correlation between various peaks and properties computed in the study, primarily relating the peaks of hexagonal ice and bulk water with computed C_V^{Levitt} and D_∞. We computed the correlation of the 4 vibrational modes of bulk water with each other and found a strong correlation between the bending ν_B and stretching ν_S vibrational peaks.

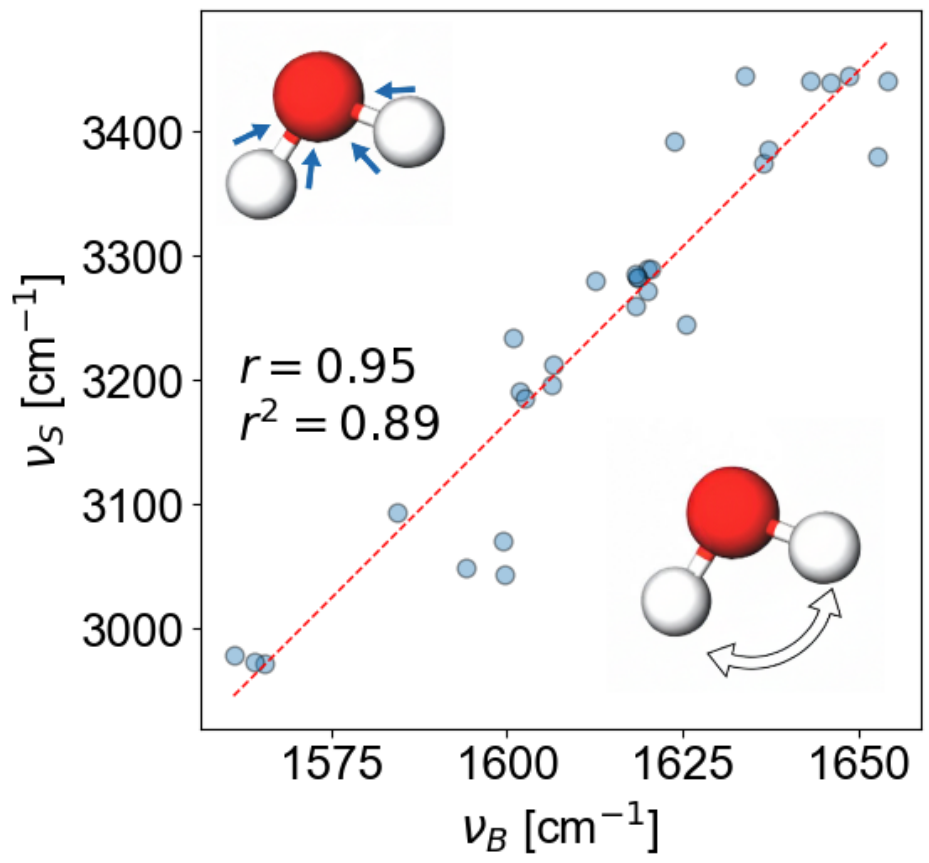

Figure S13. Correlation between vibrational peaks ν_B and ν_S peaks of bulk water.

We also assessed cross-phase predictability by studying the correlation between the libration, stretching, and bending modes of bulk water $(\nu_T, \nu_L, \nu_B, \nu_S)$ and hexagonal ice $(\nu_T^{ih}, \nu_L^{ih}, \nu_B^{ih}, \nu_S^{ih})$. This revealed strong correlations between the bending ν_B and stretching modes ν_S of bulk water with those of hexagonal ice ν_B^{ih} and ν_S^{ih}.

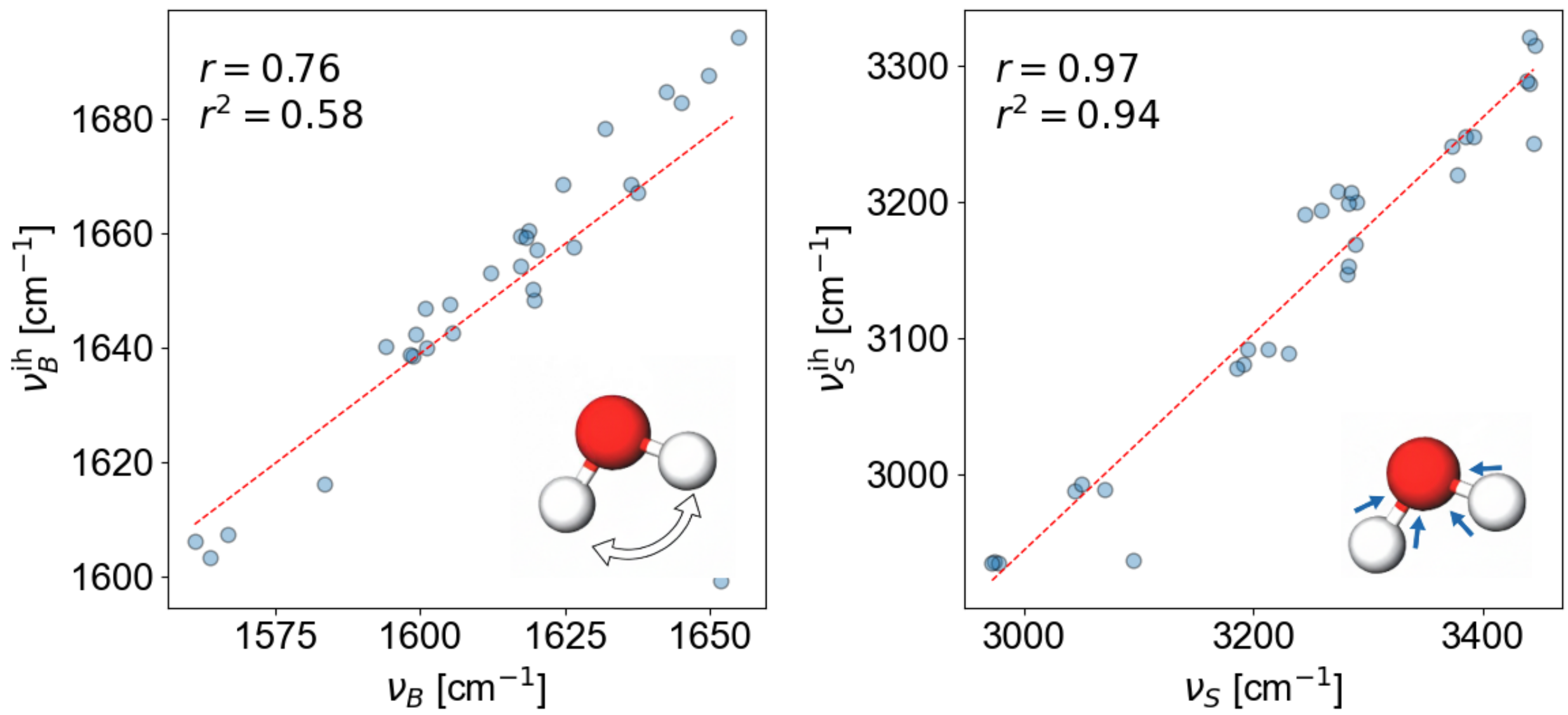

Figure S14. Correlation of bending and stretching vibrational peaks of hexagonal ice vs. bulk water.

Next, we seek to draw a structure-dynamics correlation outlined by other works [16, 22, 23] by studying the correlation of D_∞ with the first and second peaks of the oxygen–oxygen radial distribution function $g_{OO}(r_1)$ and $g_{OO}(r_2)$. We find a strong negative correlation between the peak heights $g_{OO}(r_1)$ and $g_{OO}(r_2)$ and D_∞.

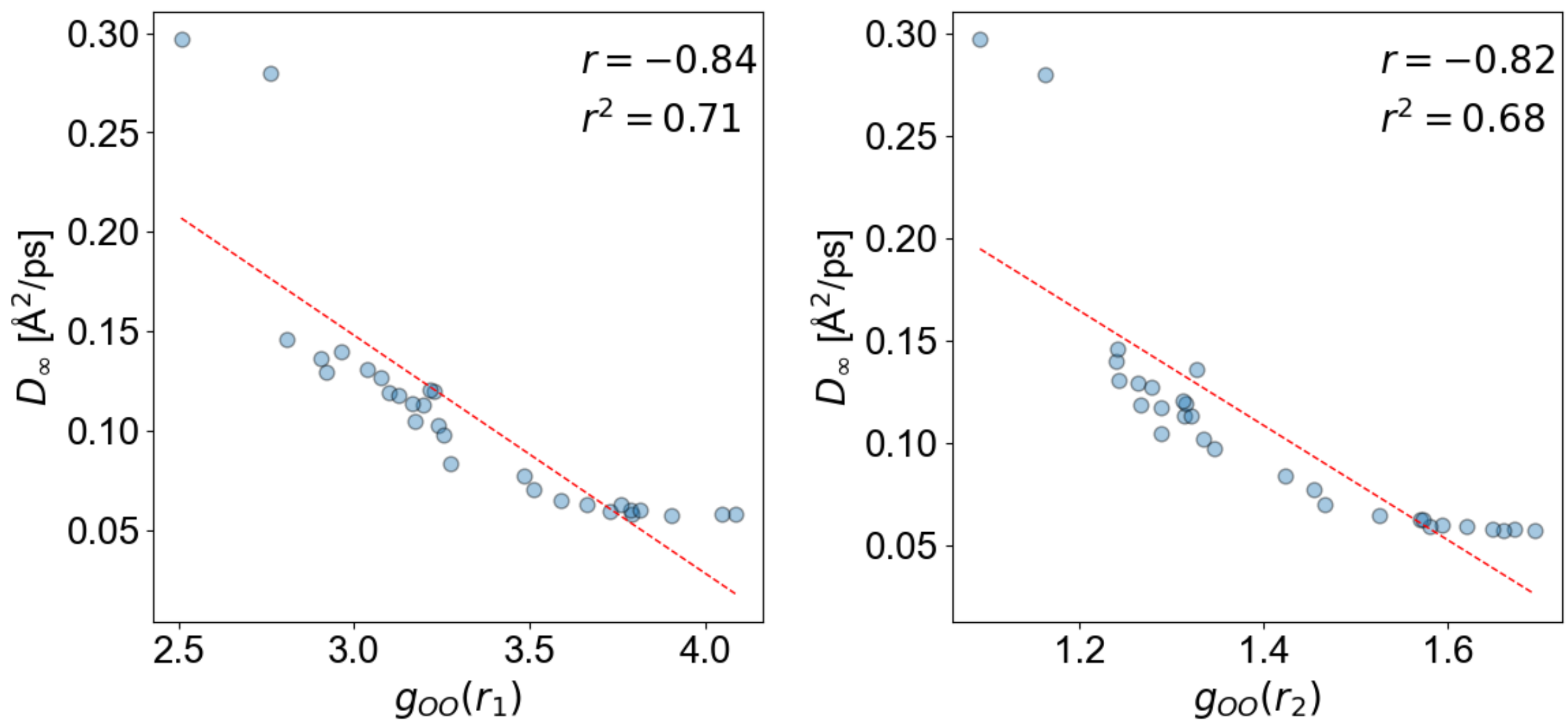

Figure S15. Correlation of $g_{OO}(r_1)$ and $g_{OO}(r_2)$ with D_∞.

[1] Michele Ceriotti, Michele Parrinello, Thomas E. Markland, and David E. Manolopoulos, "Efficient stochastic thermostatting of path integral molecular dynamics," The Journal of Chemical Physics **133**, 124104 (2010), https://pubs.aip.org/aip/jcp/article-pdf/doi/10.1063/1.3489925/13343002/124104$_1$$_o$*nline.pdf*.

[2] Mariana Rossi, Michele Ceriotti, and David E. Manolopoulos, "How to remove the spurious resonances from ring polymer molecular dynamics," J. Chem. Phys. **140**, 234116 (2014).

[3] Michael Levitt, Miriam Hirshberg, Ruth Sharon, Keith E. Laidig, and Valerie Daggett, "Calibration and testing of a water model for simulation of the molecular dynamics of proteins and nucleic acids in solution," The Journal of Physical Chemistry B **101**, 5051–5061 (1997).

[4] A.K. Soper, "The radial distribution functions of water and ice from 220 to 673 k and at pressures up to 400 mpa," Chemical Physics **258**, 121–137 (2000).

[5] Ondrej Marsalek and Thomas E. Markland, "Quantum dynamics and spectroscopy of ab initio liquid water: The interplay of nuclear and electronic quantum effects," The Journal of Physical Chemistry Letters **8**, 1545–1551 (2017), pMID: 28296422.

[6] Michele Ceriotti, Wei Fang, Peter G. Kusalik, Ross H. McKenzie, Angelos Michaelides, Miguel A. Morales, and Thomas E. Markland, "Nuclear quantum effects in water and aqueous systems," Chemical Reviews **116**, 7529–7550 (2016).

[7] John E. Bertie and Zhida Lan, "Infrared intensities of liquids xx: The intensity of the oh stretching band of liquid water revisited, and the best current values of the optical constants of h2o(l) at 25°c between 15,000 and 1 cm1," Applied Spectroscopy **50**, 1047–1057 (1996).

[8] Bernhard Schmiedmayer and Georg Kresse, "Derivative learning of tensorial quantities—predicting finite temperature infrared spectra from first principles," Journal of Chemical Physics **161**, 084703 (2024).

[9] J. E. Bertie, H. J. Labbé, and E. Whalley, "Absorptivity of ice i in the range 4000–30 cm1," The Journal of Chemical Physics **50**, 4501–4520 (1969).

[10] Mariana Rossi, Hanchao Liu, Francesco Paesani, Joel Bowman, and Michele Ceriotti, "Communication: On the consistency of approximate quantum dynamics simulation methods for vibrational spectra in the condensed phase," The Journal of Chemical Physics **141**, 181101 (2014).

[11] Daniel R. Moberg, Shelby C. Straight, Christopher Knight, and Francesco Paesani, "Molecular origin of the vibrational structure of ice ih," The Journal of Physical Chemistry Letters **8**, 2579–2583 (2017), pMID: 28541703.

[12] Shunji Imoto, Sotiris S. Xantheas, and Shinji Saito, "Molecular origin of the difference in the h–o–h bend of the ir spectra between liquid water and ice," **138**, 054506 (2013), pNNL-SA-92117.

[13] Vania Calandrini, Eric Pellegrini, Konrad Hinsen, and Gerald Kneller, "nmoldyn - interfacing spectroscopic experiments, molecular dynamics simulations and models for time correlation functions," École thématique de la Société Française de la Neutronique **12**, 201–232 (2011).

[14] Bingqing Cheng, "Cartesian atomic cluster expansion for machine learning interatomic potentials," npj Computational Materials **10**, 1 (2024).

[15] Sandeep K. Reddy, Shelby C. Straight, Pushp Bajaj, C. Huy Pham, Marc Riera, Daniel R. Moberg, Miguel A. Morales, Chris Knight, Andreas W. Götz, and Francesco Paesani, "On the accuracy of the mb-pol many-body potential for water: Interaction energies, vibrational frequencies, and classical thermodynamic and dynamical properties from clusters to liquid water and ice," The Journal of Chemical Physics **145**, 194504 (2016).

[16] Justin Villard, Martin P. Bircher, and Ursula Rothlisberger, "Structure and dynamics of liquid water from ab initio simulations: adding minnesota density functionals to jacob's ladder," Chem. Sci. **15**, 4434–4451 (2024).

[17] Tod A. Pascal, Daniel Schärf, Yousung Jung, and Thomas D. Kühne, "On the absolute thermodynamics of water from computer simulations: A comparison of first-principles molecular dynamics, reactive and empirical force fields," The Journal of Chemical Physics **137**, 244507 (2012).

[18] Luis Ruiz Pestana, Ondrej Marsalek, Thomas E. Markland, and Teresa Head-Gordon, "The quest for accurate liquid water properties from first principles," The Journal of Physical Chemistry Letters **9**, 5009–5016 (2018), pMID: 30118601.

[19] In-Chul Yeh and Gerhard Hummer, "System-size dependence of diffusion coefficients and viscosities from molecular dynamics simulations with periodic boundary conditions," The Journal of Physical Chemistry B **108**, 15873–15879 (2004).

[20] M J Holmes, N G Parker, and M J W Povey, "Temperature dependence of bulk viscosity in water using acoustic spectroscopy," Journal of Physics: Conference Series **269**, 012011 (2011).

[21] Michael Holz, Stephan R. Heil, and Antonio Sacco, "Temperature-dependent self-diffusion coefficients of water and six selected molecular liquids for calibration in accurate ^{1}h nmr pfg measurements," Physical Chemistry Chemical Physics **2**, 4740–4742 (2000).

[22] Cecilia Herrero, Michela Pauletti, Gabriele Tocci, Marcella Iannuzzi, and Laurent Joly, "Connection between water's dynamical and structural properties: Insights from ab initio simulations," Proc Natl Acad Sci U S A **119**, e2121641119 (2022).

[23] Zeke A. Piskulich, Damien Laage, and Ward H. Thompson, "A structure–dynamics relationship enables prediction of the water hydrogen bond exchange activation energy from experimental data," Chemical Science **15**, 2197–2204 (2024).
